# Cycling Empirical Antibiotic Therapy in Hospitals: Meta-Analysis and Models

**DOI:** 10.1371/journal.ppat.1004225

**Published:** 2014-06-26

**Authors:** Pia Abel zur Wiesch, Roger Kouyos, Sören Abel, Wolfgang Viechtbauer, Sebastian Bonhoeffer

**Affiliations:** 1 Institute of Integrative Biology, ETH Zurich, Zurich, Switzerland; 2 Division of Global Health Equity, Brigham and Women's Hospital/Harvard Medical School, Boston, Massachusetts, United States of America; 3 Division of Infectious Diseases and Hospital Epidemiology, University Hospital Zurich, Zurich, Switzerland; 4 Division of Infectious Diseases, Brigham and Women's Hospital/Harvard Medical School, Boston, Massachusetts, United States of America; 5 Department of Psychiatry and Psychology, School for Mental Health and Neuroscience, Faculty of Health, Medicine, and Life Sciences, Maastricht University, Maastricht, The Netherlands; University of Texas at Austin, United States of America

## Abstract

The rise of resistance together with the shortage of new broad-spectrum antibiotics underlines the urgency of optimizing the use of available drugs to minimize disease burden. Theoretical studies suggest that coordinating empirical usage of antibiotics in a hospital ward can contain the spread of resistance. However, theoretical and clinical studies came to different conclusions regarding the usefulness of rotating first-line therapy (cycling). Here, we performed a quantitative pathogen-specific meta-analysis of clinical studies comparing cycling to standard practice. We searched PubMed and Google Scholar and identified 46 clinical studies addressing the effect of cycling on nosocomial infections, of which 11 met our selection criteria. We employed a method for multivariate meta-analysis using incidence rates as endpoints and find that cycling reduced the incidence rate/1000 patient days of both total infections by 4.95 [9.43–0.48] and resistant infections by 7.2 [14.00–0.44]. This positive effect was observed in most pathogens despite a large variance between individual species. Our findings remain robust in uni- and multivariate metaregressions. We used theoretical models that reflect various infections and hospital settings to compare cycling to random assignment to different drugs (mixing). We make the realistic assumption that therapy is changed when first line treatment is ineffective, which we call “adjustable cycling/mixing”. In concordance with earlier theoretical studies, we find that in strict regimens, cycling is detrimental. However, in adjustable regimens single resistance is suppressed and cycling is successful in most settings. Both a meta-regression and our theoretical model indicate that “adjustable cycling” is especially useful to suppress emergence of multiple resistance. While our model predicts that cycling periods of one month perform well, we expect that too long cycling periods are detrimental. Our results suggest that “adjustable cycling” suppresses multiple resistance and warrants further investigations that allow comparing various diseases and hospital settings.

## Author Summary

The rise of antibiotic resistance is a major concern for public health. In hospitals, frequent usage of antibiotics leads to high resistance levels; at the same time the patients are especially vulnerable. We therefore urgently need treatment strategies that limit resistance without compromising patient care. Here, we investigate two strategies that coordinate the usage of different antibiotics in a hospital ward: “cycling”, i.e. scheduled changes in antibiotic treatment for all patients, and “mixing”, i.e. random assignment of patients to antibiotics. Previously, theoretical and clinical studies came to different conclusions regarding the usefulness of these strategies. We combine meta-analyses of clinical studies and epidemiological modeling to address this question. Our meta-analyses suggest that cycling is beneficial in reducing the total incidence rate of hospital-acquired infections as well as the incidence rate of resistant infections, and that this is most pronounced at low baseline levels of resistance. We corroborate our findings with theoretical epidemiological models. When incorporating treatment adjustment upon deterioration of a patient's condition (“adjustable cycling”), we find that our theoretical model is in excellent accordance with the clinical data. With this combined approach we present substantial evidence that adjustable cycling can be beneficial for suppressing the emergence of multiple resistance.

## Introduction

The emergence and spread of antibiotic resistance threatens our ability to treat bacterial infections and is a substantial danger for public health world-wide [1]. Resistant strains are especially prevalent in hospitals, where the high usage of antibiotics facilitates emergence and spread of resistant strains. Globally, 8% of hospital stays result in nosocomial infections [1]. It has been estimated that 70% of these are caused by single- or multiple-resistant bacteria [2]. Compared to infections by susceptible bacteria, those caused by resistant strains often increase mortality, morbidity and costs [3]. While treatment can be tailored to the pathogen and its resistance profile once cultures are available, treatment typically needs to be initiated immediately. This treatment phase is called empirical therapy. In single hospitals or wards, population-wide empirical treatment of patients can be coordinated, and several such strategies have been proposed to fight resistance [4]–[10]. Here, we focus on the comparison of two strategies on which most clinical and theoretical studies have focused so far: The first is “cycling” i.e. scheduled changes of the predominant antibiotic in a whole ward or hospital. The second is “mixing” i.e. the random assignment of patients to different antibiotics, such that at any given time point multiple antibiotics are employed in approximately equal proportions. Mixing has been seen as the strategy closest to the current usage patterns in most wards [5]. Theoretical models predict that, when different antibiotics are employed at comparable average frequencies, mixing should outperform cycling since the pathogen is subject to greater environmental heterogeneity when transmitted from host to host [5], [6]. Clinical studies addressing the general usefulness of cycling have come to contradictory results. Not only has no clear pattern emerged from these studies, but also some studies report divergent outcomes for different pathogen species. A qualitative meta-analysis [10] has argued that cycling could be beneficial for preserving drug susceptibility in *Pseudomonas aeruginosa*. However, neither a quantitative pathogen specific meta-analysis, nor a theoretical explanation of potential benefits of cycling is available to date.

Despite the difficulties to exclude confounders in the clinical setting and the often-criticized study designs [11], it may therefore be worth to re-evaluate both clinical studies and theoretical models. Specifically, it is important to elucidate whether inherent characteristics of the pathogen or the host populations may lead to these different outcomes. Here, we perform a quantitative and pathogen-specific meta-analysis of clinical studies comparing cycling to standard treatment regimens. We furthermore develop an epidemiological model tailored to the situation in hospital wards. We design this model such that it can easily describe a multitude of infectious diseases. Furthermore, we aim at a model structure that allows parameterization with observed clinical parameters. Earlier theoretical studies assumed that patients remained on the prescribed drugs until leaving the hospital (“strict cycling”/“strict mixing”). Here, we make the realistic assumption that empirical therapy is automatically changed when ineffective (“adjustable cycling”/“adjustable mixing”). For the sake of clarity, we will refer to any clinical cycling schedules as “clinical cycling”, because clinical reality is likely different from these two extremes. We compare the results of our meta-analysis with the predictions of our theoretical model and highlight common observations that may explain the divergence between earlier studies.

## Results

Unfortunately, morbidity and mortality attributable to resistant nosocomial infections are only known for a few pathogens [12]. Therefore, we need measurable proxies for disease burden. Hospital-acquired infections with both susceptible and resistant pathogens increase morbidity and mortality. In both our meta-analyses and the epidemiological model, we follow the total number of patients infected with either resistant or susceptible strains. Moreover, it has been shown that patient outcome is worse when receiving inappropriate therapy, i.e. being assigned to an ineffective initial treatment [13], [14]. In our epidemiological model, we can quantify inappropriately treated patients. However, there are no matched data for resistance profile and antibiotic therapy provided in any of the clinical studies. We chose the incidence rate of resistant infections as second endpoint in the meta-analysis, because having a resistant infection increases the probability of inappropriate therapy.

### Analysis of clinical data

Here, we define clinical cycling as repeated rotations of at least two antibiotics in the same order. We performed a literature search (see methods) to identify studies meeting these criteria. For performing a quantitative meta-analysis, we required the following additional criteria: i) a baseline period in the same ward or comparison to simultaneously recorded data from a ward in the same hospital, ii) no other infection control measures introduced in the observation period and iii) unprocessed data on the number of isolates.

As explained above, we chose the number of total isolates and resistant isolates per patient day as primary endpoints. Additionally, we collected data on mortality as a secondary endpoint. To be able to link resistance evolution to the used antibiotics and to compare the results to our epidemiological model, we only included resistance against the scheduled antibiotics. To account for multiple resistance, we summed over the number of isolates against each of the employed drugs (later referred to as weighted incidence rate of resistant infections). Moreover, we extracted data on mortality as a secondary endpoint to ascertain that cycling has no unexpected detrimental effects. We identified 46 studies [15]–[59], of which 11 were eligible [45]–[54], [59], i.e. fulfilled our criteria and provided all needed data (Figure S1, Table S1 and methods). One of these [51] reported outcomes from independent wards, which we report separately. Table S2 lists all data extracted from these studies.

**Figure 1:**
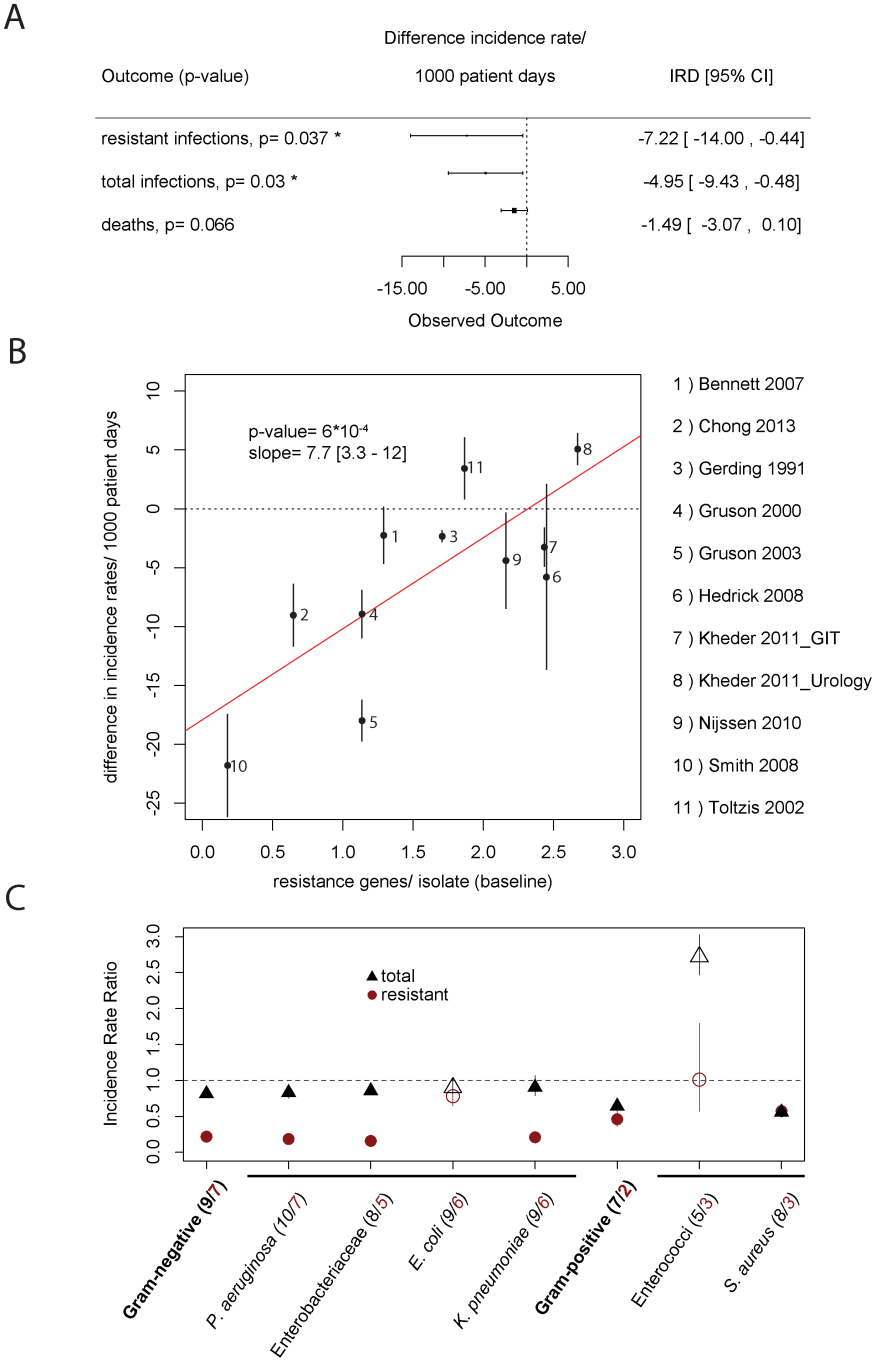
Effect of clinical cycling vs. baseline period. A) This figure shows the effect of clinical cycling on total incidence rate, weighted incidence rate of resistance, and mortality as estimated by a multivariate random-effects model. B) Performance of clinical cycling and pre-existing resistance. On the x-axis, the average number of resistances per isolate during the baseline period against antibiotics used in the clinical cycling regimen are given. On the y-axis, the success of clinical cycling as measured in the difference of total isolates per 1000 patient days is given. The error bars indicate the standard deviation for each study. The p-value as well as the slope of the regression line (red line) with 95% confidence interval of the regression is given in the figure. One study [49] was omitted because of insufficient data. C) Pathogen-specific meta-analysis. Our outcome measures were the total number of isolates (black) and the weighted prevalence of resistance to the scheduled antibiotics (red). The number of studies giving data on the respective pathogen group is given in brackets (black = total number, red = resistant). Due to the sparsity of the data for individual pathogens, we used the Mantel-Haenszel method. Empty symbols indicate pathogen groups for which the omission of one single study changed the relative benefit of clinical cycling (e.g. clinical cycling was beneficial when all studies were considered, but the omission of one of these studies led to a detrimental outcome or vice versa).

For all endpoints, cycling performed significantly better in univariate analyses, also after adjustment for multiple testing (Table S3). However, the three endpoints are correlated (not independent), such that univariate meta-analyses on each individual endpoint is inferior to a multivariate meta-analysis on those three endpoints simultaneously. We employed a multivariate analysis framework (methods and supplementary materials p. 9), which revealed significant reductions in the weighted incidence rate of resistant isolates from 27 to 20 isolates/1000 patient days (p = 0.037) as well as in the total incidence rate from 30 to 25 isolates/1000 patient days (p = 0.03, Figure 1A).

We found a pronounced correlation (p = 0.00059, p = 0.028 after Benjamini-Hochberg correction for multiple testing) between the total incidence rate and level of resistance against the cycled drugs during the baseline period as measured in average number of resistances per isolate (Figure 1B). At low levels of baseline resistance, clinical cycling reduced the total incidence rate of resistant infections substantially.

Because of the enormous biological differences between the various bacterial pathogens that may cause hospital-acquired infections, we repeated our analysis for single pathogen groups and species (Figure 1C). While clinical cycling remains overall beneficial, its success strongly depended on the pathogen species.

Differences in antibiotic consumption and import of resistance into the hospital are strong confounders when comparing strategies to fight resistance. It has e.g. been argued that conducting a study per se might alter prescription behavior and thus reduce antibiotic usage and increase antibiotic heterogeneity [60]. We collected data on overall antibiotic heterogeneity during baseline and clinical cycling (in this case measured over all periods), antibiotic usage and whether the study controlled for imported pathogens. We obtained very similar estimates for the effect of clinical cycling (Table 1) when adjusting for these three confounders in a multivariate meta-regression. A sensitivity analysis of the results can be found in the supplementary material (text S1, †table S4, S5).

**Table 1:** Outcomes adjusted for confounders.

Endpoint	Adjusted for antibiotic usage and heterogeneity	Adjusted for import into hospital
difference total incidence rate/1000 patient days	−7.5 [−12.3, −2.6]	−9 [−18.5, 0.4]
difference resistance incidence rate/1000 patient days	−10.0 [−20.3, 0.2]	−3.0 [−15.3, 9.3]
difference deaths/1000 patient days	0.4 [−2.6, −3.5]	−2.6 [−5.9, −0.7]

This table shows how the obtained results are affected by commonly criticized confounders. To adjust for differences in antibiotic usage and heterogeneity, we predicted new estimates for univariate with the function predict() as implemented in the package metafor in R. Here, antibiotic heterogeneity is defined with the antibiotic heterogeneity index, 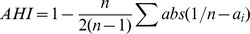 with n = number of employed antibiotics and a_i_ = usage of antibiotic a/total antibiotic usage.

To adjust for antibiotic heterogeneity and consumption, we predicted the estimates for the hypothetical case that the ratio of daily defined doses and antibiotic heterogeneity indices is 1, i.e. exactly equal antibiotic consumption and heterogeneity in both study arms. For predicting the difference when controlling for hospital import, we predicted estimates for the hypothetical case that all studies only report isolates of strains that the respective patients were neither colonized nor infected with at admission.

### Theoretical model

We used our epidemiological model to investigate whether we could find theoretical explanations for the results of our meta-analysis. Unlike previous work, our model distinguishes between asymptomatically colonized and symptomatically infected patients. In particular, we make the realistic assumption that treatment is adapted if an asymptomatically colonized patient progresses to symptomatic disease. For instance, patients receiving drug A are switched to drug B when their condition deteriorates. In clinical practice, it may be impossible to adhere to the current treatment regimen under all circumstances. To accommodate for variable adherence, we include patients treated with neither of the scheduled drugs as well as patients treated with both drugs simultaneously. We also consider two transmission modes: delayed transmission via contaminated surfaces and direct transmission. Furthermore, we consider a stochastic and a deterministic version, which describe small populations (i.e. single wards) and large populations (i.e. entire hospitals), respectively.

**Figure 2:**
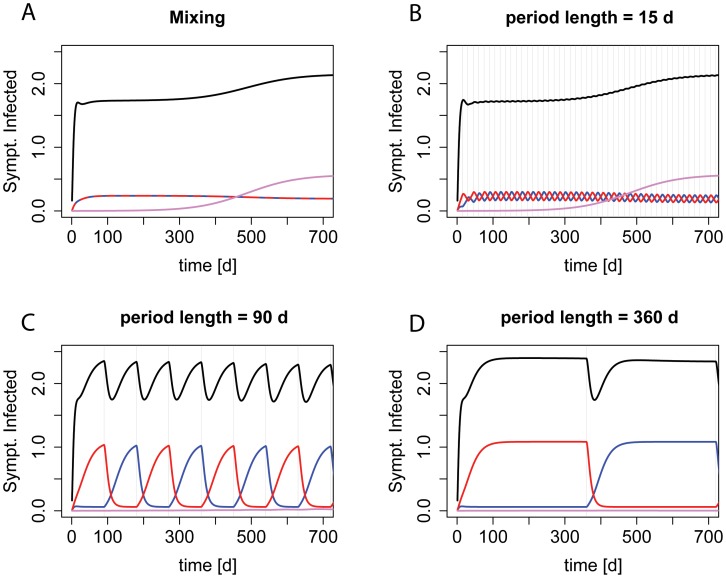
Dynamics during “adjustable mixing” and “adjustable cycling” periods of different length. The prevalence of symptomatically infected patients by strain genotype during deterministic realizations of scenario ii) (single-resistance among incoming patients) are shown. Red indicates resistance to drug A, blue indicates resistance to drug B, purple resistance to both drugs, and black the overall prevalence. Graph A) shows the dynamics during mixing, B)–D) during cycling with increasing period length. The grey vertical lines indicate a period change.

We employed our model to address how the benefit of “adjustable cycling” changes with the period length. Figure 2 gives an overview of the dynamics during different period lengths.

**Figure 3:**
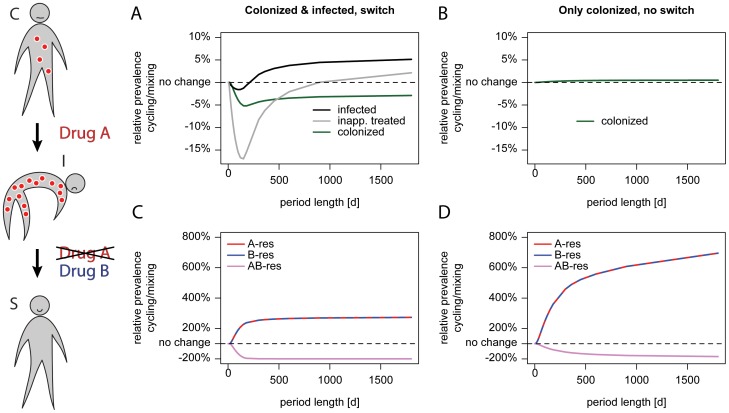
Cycling is successful when treatment is adjusted. The schematic on the left illustrates treatment adjustment upon progression. A patient colonized (C) with an A-resistant pathogen as indicated by red circles and receiving drug A progresses to symptomatic disease (I) because the current drug is ineffective. Upon progression, the therapy under which the patient deteriorated is switched to drug B. This drug is effectively clearing the infection and the patient becomes susceptible (S) for new colonization. The left panel (A, C) shows a scenario where treatment is adjusted upon progression, the right panel (B, D) shows the same parameter without treatment adjustment. We modeled the scenario without treatment adjustment by setting the progression rate to zero, the treatment frequencies for colonized patients were adjusted to correspond to the overall treatment frequencies in our standard settings. The x-axis gives the period length, the y-axis the prevalence of single-resistant carriers relative to Mixing. The upper panel (A, B) shows the prevalence of colonized (green), symptomatically infected (black) and symptomatic patients who are inappropriately treated (grey). The lower panel (C, D) shows the genotype composition depending on the period length: red indicates resistance to A, blue resistance to B, and purple resistance to both drugs. The dotted black line indicates no difference in prevalence.

For the extremes of the screened period range, our findings are in accordance with previous studies (Figure 3, S2, and S3). For periods below 5 days, there is no difference between “adjustable cycling” and “adjustable mixing”. Intuitively, this makes sense because for cycling periods below the average length of stay (in our standard setting 6.8 days), at a given time point the patients in the ward will have started their therapy in different phases of the cycling regime, and will therefore be treated with different drugs. Thus, “adjustable cycling” leads to a similar heterogeneity in drug use as “adjustable mixing”. For very long periods, “adjustable cycling” performs worse than “adjustable mixing”. This is because cycling with long periods is almost equivalent to strict mono-therapy, leading to high frequencies of the currently favored single-resistant strain (Figure 3D, S2, and S3). However, we find for most parameter settings that “adjustable cycling” outperforms “adjustable mixing” for a range of intermediate periods (Figure S4).

The success of “adjustable cycling” might be attributable to extinction of strains that are resistant to the antibiotic that is currently unused. Surprisingly, adjustable cycling performs worse in stochastic models, falsifying that hypothesis (Figure 3, S2, and S3). Our findings are in contrast to earlier studies that employed deterministic models [5], [6], which have argued that cycling always performs worse than mixing. In particular, it was argued that the disadvantage of cycling is monotonically increasing with the cycling period [5].

We tested which of our model assumptions changes the predictions so fundamentally. Unlike previous work, our model distinguishes between asymptomatically colonized and symptomatically infected patients. In particular, we make the realistic assumption that treatment is adapted if an asymptomatically colonized patient progresses to symptomatic disease. For instance, patients receiving prophylaxis with drug A are switched to drug B upon progression. We compared our chosen endpoints as well as the prevalence of different genotypes either with (Figure 3 A and C) or without (Figure 3 B and D) this adjustment of treatment. When assuming that there was no progression from colonization to symptomatic infection, the number of colonized patients raised monotonically with the period (Figure 3 B) as observed by Bergstrom et al. [5]. Why does an adjustment of therapy make “adjustable cycling” effective? The most pronounced difference is that the prevalence of singly resistant pathogens is lower with the adjustments (Figure 3 C and D). This is because the treatment switch slows the rise of those pathogens, which are resistant to the current treatment during a particular cycling period. At the same time, a substantial amount of resistant infections is washed out by asymptomatic carriers leaving the hospital. Due to this fast decline and the slow rise of resistance, a previously restricted drug can be successfully re-employed in the next cycling period.

As mentioned above, one aim of this study was to investigate whether we could find theoretical explanations for potentially divergent recommendations depending on hospital settings or differences in pathogen biology. To this end, we screened a very large parameter space (see material and methods and text S1). We defined the optimal period as the period most successfully reducing inappropriate treatment without leading to an increased prevalence of symptomatic infections (Figure S4). A sensitivity analysis describing how the optimal period can be found in the supplementary material (Figure S5, S6, and S7). In our model, “adjustable cycling” with a fixed period of 30 days was often successful and rarely clearly disadvantageous. Despite enormous improvements in some settings when individually adjusting the period for each setting, the optimal period only performed 1.6% (median) better than a fixed period of 30 days. A detailed analysis of the influence of specific disease and hospital characteristics is given in the text S1. In accordance with the results of our meta-regression, adjustable cycling is especially advantageous if multiple resistance has not risen to high frequencies yet but is likely to rise further.

## Discussion

The question when to use cycling or mixing has been controversially debated [10], and clinical and theoretical studies came to different conclusions [5]–[7], [9], [10], [61]. There are two possible explanations for this divergence between theory and clinical observations and potentially between different pathogens: We might not have sufficient data on population-wide resistance emergence. Alternatively, specific settings or differences in pathogen biology might not have been adequately captured in theoretical models so far.

We employed a method for a quantitative multivariate random-effects meta-analysis using incidence rates as endpoints and find that cycling reduced the incidence rate for both total infections and resistant infections. Our findings remain robust in uni- and multivariate metaregressions.

We investigated the influence of 60 factors that might affect study outcome by meta-regressions. Regressions with a single potential confounder are simplifications and might bias our results. However, if the results of the meta-regressions and the mathematical model are in concordance, it is justified to suggest that the tested factors influence the success of cycling. Indeed, we found that clinical cycling reduced the number of total infections when the pathogens isolated during the baseline period had a low average number of resistance genes to the drugs employed in the cycling regimen. This is in line with our and previous theoretical results arguing that cycling is effective in preventing the evolution of multiple resistance, but that there is little difference once multiple resistance is wide-spread [5], [6], [62].

In our analysis, we assume that the baseline period most closely resembles “adjustable mixing”. However, physicians might tend to prescribe drugs that both cover a broad spectrum of pathogens and which they are familiar with. Additionally, they may be asked to use the substance that has the lowest cost. These restrictions may lead to a predominance of one drug. Furthermore, there is only one clinical study simultaneously comparing mixing and cycling [63]. Finally, the implementation of a study might alter prescribing behavior [60]. We examined these confounders by adjusting for different AHIs (antibiotic heterogeneity indices) [28], as well as the total volume of antibiotic consumption (measured in daily defined doses, DDD). These adjustments did not generally change the outcome.

Another potential confounder may be differences in the influx of resistant pathogens into the hospital. Some of the studies we analyzed controlled for this confounder. Additional adjustment for the differences in these two study groups further widened the confidence interval such that all differences became statistically insignificant, while the general trend towards a positive effect of clinical cycling remains. Although adjustment leads to less clear results, we would expect that confounders are comparable for all considered pathogens and confounders therefore do not explain the divergent findings for different pathogens. Moreover, the adjusted endpoints are not uniformly shifted towards a less favorable outcome. This indicates that the observed success of clinical cycling is not solely attributable to confounders that were most criticized [11]. Despite testing for publication bias (data not shown), we cannot exclude that unsuccessful studies were not published. However, we chose a new composite outcome (weighted incidence rate of resistance), making publication bias for this particular measure less likely.

Nosocomial infections can be caused by a large variety of bacterial pathogens. We therefore repeated our analysis for important pathogen groups and bacterial species. Again, the overall effect of clinical cycling was beneficial, especially in reducing resistance. Surprisingly, we observed large differences between different pathogens. The detrimental effect of clinical cycling regarding infections caused by enterococci might be partially explained by the fact that one study used linezolid and vancomycin for their regimen, leading to outbreaks of vancomycin-resistant enterococci (VRE) in the vancomycin periods [54]. Also from previous theoretical studies as well as our theoretical results, we expect that cycling favors singly-resistant pathogens.

The study design of many clinical studies had been criticized before [11]. Based on the available studies that fulfill our selection criteria, our meta-analyses consistently show that clinical cycling is beneficial. To strengthen this finding, we used mathematical modeling to investigate the underlying mechanisms that explain our results. This theoretical epidemiological model specifically addresses the situation in hospitals and can be adapted to a multitude of infectious diseases. Importantly, our model allows adjusting ineffective treatment. These “adjustable strategies” are different from the strict cycling and mixing modeled in previous theoretical work, but is likely to be closer to clinical reality.

The flexibility of our model enables us to identify the optimal period for a large parameter-range for several settings. This is essential for elucidating the influence of pathogen biology on optimal treatment strategies. Our model includes many of the characteristics of hospital wards that were not considered in previous models. However, we made simplifications that are discussed below. These simplifications were necessary because screening a large parameter space would become impossible with increasing model complexity. Importantly, for parameter settings corresponding to those in earlier models we come to the same conclusions.

We assume that resistance always fully protects from the effects of the antibiotics and neglect any within-host dynamics. We only model a single hospital ward and assume that the composition of incoming patients is constant. In our modeling framework, the susceptible state is a result of previous antibiotic therapy. It indicates that a patient's microflora has been disturbed to a degree that other strains can easily invade. Despite the large numbers of bacteria in the microflora in colonized patients, their numbers likely decline when treated with an antibiotic for which they are susceptible. This process would be most accurately described by a continuous decline of infectiousness, which may never reach zero. However, to keep our model tractable, we assume that the bacterial load in the microflora is reduced to an extent that transmission of a strain susceptible to the used antibiotics is negligible compared to the infectious pressure by fully colonized or infected patients.

Furthermore, we assume that the mutation rates to resistance are constant. With plasmid-borne resistance, the rate of resistance acquisition depends on the abundance of both donor- and acceptor strains. Thus, our model reflects chromosomal resistance more accurately than plasmid-borne resistance. However, mutation rates have a negligible influence once resistance is brought in by incoming patients. We therefore do not expect that the results of our model would change substantially when taking different modes of resistance acquisition into account.

Naturally, there is an enormous biological diversity in all pathogens that cause nosocomial infections. Thus, we would expect differences in the speed of resistance emergence and spread. Here, we focus on the question, which salient properties of these bacteria determine which treatment strategy will be most successful. One important factor we identify in all our analyses is the rate of emergence of multiple resistance. In our general meta-analysis we found that the baseline prevalence of resistance strongly affects the success of cycling. Consistent with these results, we observe in our model that “adjustable cycling” can suppress the emergence of multiple resistance. This is the case when multiple resistance is not present in incoming patients, but would emerge *de novo* in the ward during “adjustable mixing”, i.e. with high mutation rates in the stochastic model and more generally in the deterministic model. The fact that “adjustable cycling” is even more effective in a deterministic model indicates that extinction events during the off-periods play only a minor role and cannot explain potential advantages of cycling. This is in contrast to making use of extinction in informed switching, where treatment is switched depending on current resistance frequencies [64]. Unsurprisingly, these results only hold when the single-resistant strains have a competitive advantage over the double-resistant strain in each cycling period.

The optimal period depends on the emergence of double-resistant strains and the generation time (time between the infection of a patient and the transmission to the next patient). These factors are not always known, but a period length of 30 days performed well in nearly all settings. When in doubt, a shorter period seems to be more beneficial, because there is no difference between “adjustable cycling” and “adjustable mixing” when the period length is shorter than the generation time, while too long periods are equal to treating with only one drug. Despite the lack of correlation between number of used drugs and study outcome in the meta-analysis, it would also be interesting to develop theoretical models with more than two drugs. From previous theoretical studies [6], we would expect that cycling improves as more drugs are included, because resistance against a specific drug would decline to lower levels until this drug is reintroduced.

Most importantly, the findings of our meta-analysis agree well with our theoretical results. Both the meta-analysis as well as the theoretical model shows that cycling is beneficial if there is emerging or a low influx of double-resistance. Thus, our model incorporates an important, previously disregarded factor that changes treatment recommendations. Clearly, more pathogen-specific studies of larger scales are needed to answer in which pathogens cycling is beneficial.

## Methods

### Meta-analysis

#### Study selection

A literature search was performed on PubMed in the Medline database using ‘hospital” combined with two of the following terms: i) ‘antibiotic’, ‘antimicrobial’ or ‘antibacterial’ and ii) ‘cycling’, ‘rotation’ or ‘scheduled changes’. For a search on Google Scholar we required i) and ii) to be linked, i.e. either *“i) ii)”* or *“ii) of i)”* to minimize false-positive hits in the full text search. Reference lists of all retrieved original papers and of review articles were hand-searched to identify further relevant studies. We identified 46 clinical studies addressing the effect of cycling on nosocomial infections (Figure S1).

#### Inclusion/exclusion criteria

We defined cycling as repeated rotations of at least two antibiotics in the same order. This criterion was met by 25 studies (Figure S1) Furthermore, we required a baseline period in the same ward or comparison to simultaneously recorded data from a ward in the same hospital and that no other infection control measures were introduced in the observation period. In total, 11 studies were eligible, i.e. fulfilled our criteria and provided all needed data (Figure S1).

#### Endpoints

We chose the number of total isolates and resistant isolates per patient day as primary endpoints and deaths/patient day as secondary endpoint. To be able to compare the results to our model, we only included resistance against the scheduled antibiotics and summed over all resistances, such that we count resistance genes rather than the number of isolates resistant against at least one drug. This measure is related to the number of inappropriately treated patients, since appropriate treatment becomes less likely with increasing resistance levels. Since not all studies gave the number of patient days, we used the number of beds in ward or in the hospital multiplied with observed period as a proxy when necessary.

#### Data extraction

Data were extracted independently by two investigators (PAzW and SA). Differences were resolved by discussion with a third investigator (RK). The extracted data are given in table S2. For one study [45], both a temporal and a spatial control were given. Three studies reported both total and only acquired pathogens [11], [50], [54]). To minimize heterogeneity, we chose the temporal control and total isolates. However, when adjusting for possible confounding (Table 1), we used the difference in the three studies reporting both total and acquired pathogens. Regarding antibiotic usage, the level of detail in reporting was very variable. To calculate antibiotic heterogeneity ([28], Table 1), we used all data reported in each study regardless of how comprehensive they are.

#### Data analysis and statistical methods

We used the metafor package [65] in the statistical software package R (version 3.0.2). For analyzing the incidence rates of all reported pathogens, the pooled rate differences and 95% confidence intervals (CIs) were calculated using data provided in each study. For the data on infections caused by all bacterial species, these were obtained using a random-effects multivariate meta-analysis of the three endpoints simultaneously ([66]–[68] see supplementary material).

Since the data for single pathogens are sparse, we used the Mantel-Haenszel method and evaluated the stability of the results with leave-one-out sensitivity analyses. Between-study heterogeneity was examined using the Q statistic and the I2 statistic [69]. Publication bias was assessed using plots of study results against precision of the study (funnel plots). Symmetry of the funnel plots was tested using the methods suggested by Egger et al. [70] and Begg and Mazumdar [71]. Given the detected high degree of heterogeneity of the incidence rate differences, we subsequently conducted meta-regression analyses to explore pre-defined sources of heterogeneity.

### Epidemiological model

We assess the outcome for a timeframe of ten years to account for the fact that the expected time for the availability of new broad-spectrum antibiotics is in the range of a decade. We performed all analyses with a stochastic and a deterministic version, which describe small populations (i.e. single wards) and large populations (i.e. entire hospitals), respectively. In preliminary analyses, we found that transmission mode and the proportion of incoming resistant strains were the factors that lead to the greatest changes in model predictions. Therefore, we chose a total of six standard scenarios. We consider two transmission modes: delayed transmission via contaminated surfaces and direct transmission. Both transmission modes were analyzed for three settings: either i) no pre-existing resistance in the community, ii) pre-existing single-resistance or iii) both single and double-resistance pre-exist. For these six standard scenarios, each of the 22 model parameters was varied over a clinically relevant range (Table S6), while all other parameters were kept at default values. All periods were chosen such that we evaluate the success at 3600 days exactly at the end of a period where the second antibiotic (B) was employed. For all six settings, we screened the combination of all default values by varying the period length over all integer divisors of 1800 (i.e. 1800, 900, 600, …, 2, 1). When varying single parameters in each of the six standard settings, we chose a subset between 5 and 360 days (Figure S4).

The model we use in this study is based on a model we used previously [64], [72]. We consider a compartmental epidemiological model that aims at describing a single hospital ward (for an overview over the parameterization see Table S6). We assume that two broad-spectrum antibiotics are available for empirical treatment. We will refer to these as drug A and B. Accordingly, we follow four genotypes (Figure S8): wild type (sensitive to both drugs), resistant to A, resistant to B, and resistant to both drugs. Resistance can be acquired via mutations, which occur at rates *μ*_a_, *μ*_b_ and *μ*_ab_, the subscript denotes the drug against which resistance is acquired. The parameter *μ*_asym_ describes *μ*_b_ relative to *μ*_a_, while keeping the resulting *μ*_ab_ constant. For simplicity, we make the assumption that there is no cross-resistance, meaning there is no additional selection pressure for A-resistance or B-resistance other than by drug A and B, respectively.

**Figure 4:**
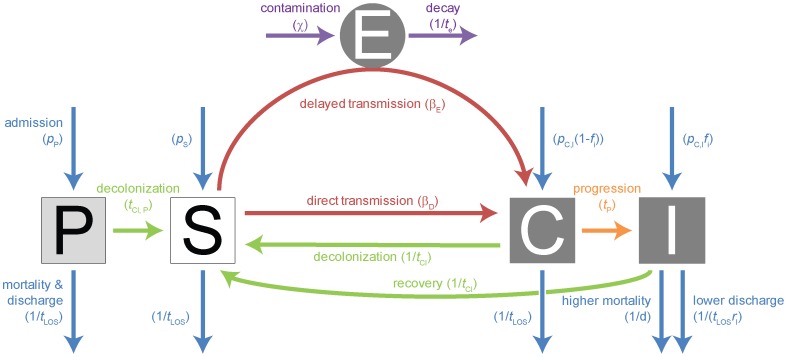
Compartmental model for single strain. Explanations of the parameters, their standard values, the range over which we varied these parameters, as well as references are given in table S6. The compartments are: P = protected patients; S = susceptible patients; C = colonized patients; I = infected patients; E = environment. The color coded arrows indicate: violet = environmental contamination & decay; blue = admission & mortality/discharge; green = decolonization & recovery (this does not necessarily indicate full clearance of the pathogen from all body compartments, rather, it describes that the bacterial population has decreased sufficiently to allow a new strain to take over); orange = progression; red = transmission.

Patients are classified as being protected (P, e.g. intact microflora), susceptible (S), colonized (C; i.e. asymptomatic carriers), or infected (I; i.e. symptomatic carriers) (Figure 4). In this context the susceptible state is a result of previous antibiotic therapy. It indicates that a patient's microflora has been disturbed to a degree that other strains can easily invade. Furthermore, we assume that the bacterial load in the microflora is reduced such that transmission of a strain susceptible to the used antibiotics ceases.

We assume that both mortality and morbidity only differ in symptomatically infected patients, the additional mortality in these patients is given by the parameter *d*, the reduced likelihood of leaving the hospital when infected by the parameter *r*_I_. With long-term treatment, protected patients may proceed to the susceptible compartment after a time *t*_cl,P_. We consider two transmission modes; either immediate transmission (also appropriate for transmission without a time-lag via health care workers) or delayed transmission. The latter occurs via a pathogen reservoir outside the patients (E), which describes most appropriately environmental contamination. It may also describe the dynamics resulting from the transient colonization of health care workers, although these are not modeled explicitly. Patients are first asymptomatically colonized and may then progress after a time *t*_p_. The time to clearance *t*_cl_ when treated appropriately is the same for both colonized and infected patients. The compartments C and I are divided in subcompartments according to the carried genotype (wt, A-, B- or AB-res).

**Figure 5:**
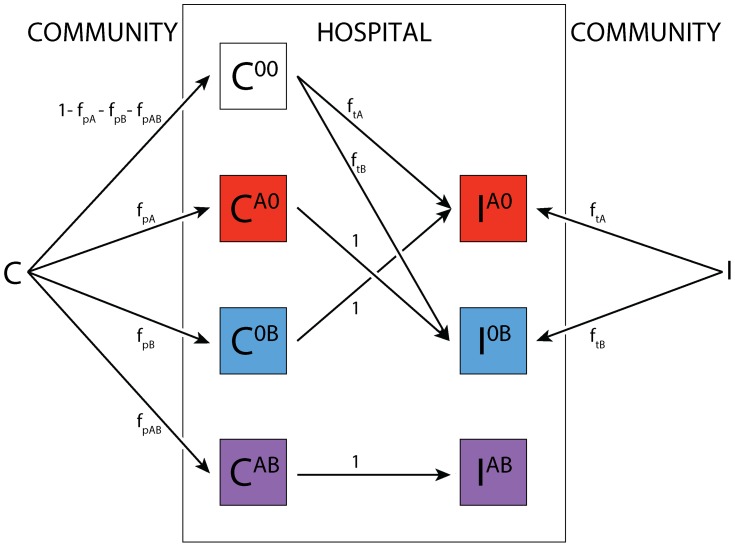
Treatment algorithm. The superscript denotes the treatment status. Colonized patients are assigned with frequencies *f*_p_ and *f*_p,AB_ to treatment for other causes than symptomatic infections with the organism under consideration. If they progress to symptomatic disease and were previously treated with a single drug, this drug is then switched, while patients on both drugs remain on their treatment. Infected patients are assigned to treatments according to the current treatment strategy (mixing or cycling) immediately upon entering the ward.

We assume a fixed number of beds (20) in the hospital ward. As soon as a bed is free, new patients are admitted within a day, resulting in an average population size of ∼17 patients per ward (85% occupancy). The composition of the incoming patients regarding colonization and resistance status is assumed to be constant over the observed timeframe. These frequencies are described using the parameters in table S6, section 2. The proportion of patients carrying resistant and double-resistant strains is given by *p*_res_ and *p*_ab_, respectively, the relative proportions of A- and B- resistance are given by *p*_asym_ (if this is 0.5, both strains are found at equal frequencies). To follow treated patients, all compartments are subdivided according to the treatment status (Figure 5). Since we only investigate resistance to drug A and B, we do not take any other drugs into account. Infected patients are treated per default according to the current treatment strategy (“adjustable mixing” or “adjustable cycling”) as soon as they enter the hospital or progress to the infected compartment.

Here, we consider “adjustable strategies”, i.e. we assume that in patients that progress while they are treated, the treatment is switched. Furthermore, we assume that susceptible patients cannot be infected with a strain when treated with a drug the pathogen is susceptible for. A certain fraction of patients that are not symptomatically infected with the considered pathogen may also receive treatment with the scheduled drugs. The frequency of such treatment, which we call here prophylaxis, is given by *f*_p_, and describes the number of asymptomatic patients receiving the currently scheduled therapy. In addition, some patients may receive both drugs, this is denoted by *f*_p,AB_. Figure S9 gives the average treatment frequency during one strategy. Around seven of seventeen patients (41%) receive either of the scheduled drugs at any point in time. Once an infected patient is assigned to a drug, he remains on this treatment until he leaves the hospital unless the treatment is inappropriate (for example treatment with drug A for an A-resistant infection), in which case it is switched with a rate *s*.

The biology of different infectious diseases is described with the parameters of table S6, section 3. These determine a) how fast de novo resistance may arise in individual patients, b) the costs of these resistance mutations, which are assumed to lower transmission probability, c) the rates with which patients can recover after treatment, d) the increase in mortality by the disease and finally e) how fast colonized patients become symptomatic.

We consider both a deterministic and a stochastic version of the model described above. The deterministic model is implemented by numerically solving the ordinary differential equations (ODEs) that correspond to figures 4, 5 and S8. The stochastic model is derived from these ODEs by considering transition between compartments as stochastic events according to the Gillespie algorithm. All codes are available upon request.

## Supporting Information

Figure S1 **PRISMA flowchart.**
(PDF)

Figure S2 **Influence of period length on “adjustable cycling” success and genotype composition.** Results of stochastic simulations with parameters for scenario ii (single-resistance present among incoming patients) and direct transmission. **A**) Relative change of inappropriately treated patients (open grey diamonds) and symptomatically infected patients (solid black circles) as compared to “adjustable mixing”. **B**) same as A with higher resolution. **C**) Relative change of genotype composition depending on the period length as compared to “adjustable mixing”: black indicates wild-type, red resistance to A, blue resistance to B, and dotted purple resistance to both drugs. **D**) same as C) with higher resolution. The 95% CI, as determined by bootstrapping, is given as error bars. Please note that the error bars for all measures except the prevalence of double-resistance are smaller than the used symbols.
(PDF)

Figure S3 **Influence of period length on cycling success and genotype composition, environmental transmission.** For all simulations, parameters for scenario ii (single-resistance present among incoming patients) and environmental transmission were used. **A**) Relative change of inappropriately treated patients (open diamonds) and symptomatically infected patients (solid circles) as compared to mixing for a deterministic realization. **B**) Same as A) for a stochastic realization. **C**) Relative change of genotype composition depending on the period length as compared to mixing: black indicates wild-type, red resistance to A, blue resistance to B, and dotted purple resistance to both drugs. The 95% CI, as determined by bootstrapping, is given as error bars. **D**) Same as C) for a stochastic realization.
(PDF)

Figure S4 **Overview over optimal cycling periods and success of these periods.** **A**) For stochastic simulations if no resistance pre-exists in the incoming patients (scenario i). The results were obtained by averaging over 10000 simulations. Parameters (explained in table S6) were varied from a) (lowest) to g) (highest, see table S6) and the effect on the relative reduction of inappropriately treated patients as compared to mixing is indicated by green color code for an optimal period length. The optimal period was defined as the period that is most successful in reducing inappropriate therapy without leading to a higher prevalence of symptomatic infections and its length is indicated by red color code. The areas shaded in grey indicate that there is no period (within our screened range) for which cycling outperforms mixing. **B**) For stochastic simulations if only single-resistance pre-exists in the incoming patients (scenario ii). The results were obtained by averaging over 10000 simulations. Parameters (explained in table S6 were varied from a) (lowest) to g) (highest, see table S6 and the effect on the relative reduction of inappropriately treated patients as compared to mixing is indicated by green color code for an optimal period length. The optimal period was defined as the period that is most successful in reducing inappropriate therapy without leading to a higher prevalence of symptomatic infections and its length is indicated by red color code. The areas shaded in grey indicate that there is no period (within our screened range) for which cycling outperforms mixing. **C**) For stochastic simulations if both single and double-resistance pre-exist in the incoming patients (scenario iii). The results were obtained by averaging over 10000 simulations. Parameters (explained in table S6) were varied from a) (lowest) to g) (highest, see table S6 and the effect on the relative reduction of inappropriately treated patients as compared to mixing is indicated by green color code for an optimal period length. The optimal period was defined as the period that is most successful in reducing inappropriate therapy without leading to a higher prevalence of symptomatic infections and its length is indicated by red color code. The areas shaded in grey indicate that there is no period (within our screened range) for which cycling outperforms mixing. **D**) For deterministic simulations if no resistance pre-exist in the incoming patients (scenario i). Same as Figure S4A for deterministic simulations. **E**) For deterministic simulations if single-resistance pre-exists in the incoming patients (scenario ii). Same as Figure S4B for deterministic simulations. **F**) For deterministic simulations if both single and double-resistance pre-exist in the incoming patients (scenario iii). Same as Figure S4C for deterministic simulations.
(PDF)

Figure S5 **Influence of admission of susceptible patients and time between first and second transmission.** The lines represent the averages of 10000 stochastic simulations; the error bars the 95% CI as determined by bootstrapping. The red line indicates the standard parameter setting. **A**) Shows an example of increasing the influx of susceptible patients (from 0%, given in black in 15% steps to 90%, given in light grey). Simulations for direct transmission for scenario ii (only single-resistance among incoming patients) are shown. **B**) Shows an example of increasing the time between the colonization of a patient and the transmission of the pathogen by increasing the environmental decay rate (from 1 day, given in black over 2, 5, 7, 10, 20 to 100 days, given in light grey). Simulations for environmental transmission for scenario ii (only single-resistance among incoming patients) are shown. The dotted black line indicates no difference in prevalence.
(PDF)

Figure S6 **“Adjustable cycling” success depends on fitness of double-resistant strain.** These graphs show how the suppression of double-resistance depends on the fitness of the double-resistant strain (color code on the right). The parameter setting corresponds to scenario ii (single-resistance among incoming patients) for direct transmission. The upper panel (A, B) gives the prevalence of inappropriately treated patients relative to mixing; **A**) for deterministic realizations and **B**) for the stochastic realizations. The 95% CI, as determined by bootstrapping, is given as error bars, however, these are smaller than the line width. The lower panel (C, D) gives the prevalence of double-resistant strains relative to mixing; **C**) for deterministic realizations and **D**) for the stochastic realizations. The 95% CI, as determined by bootstrapping, is given as error bars.
(PDF)

Figure S7 **Selection pressure depending on period length and main factors influencing the optimal period.** **A**) Classification of period lengths in three groups, on the example of a stochastic realization of the standard setting for scenario ii (single-resistance present among incoming patients). i) red indicates those periods for which there is no difference between “adjustable mixing” and “adjustable cycling”, ii) green indicates those periods, which select against single-resistance and for double-resistance, iii) black those that both increasingly select against double-resistance and increasingly allow outgrowth of single-resistant strains. B) to D) show examples of the main factors influencing the location and success of the optimal period, the arrows summarize the trends which occur by changing these factors. **B**) Influence of the difference between the prevalences of the single resistant strains during the on- and off-periods in “adjustable cycling”. The black line indicates the standard value as in A), the grey line indicates a setting with more incoming susceptible patients **C**) Influence of the turnover rate on the optimal period. The black line indicates environmental transmission with a decay rate of one day (note the similarity to the standard setting for direct transmission), the grey line indicates environmental transmission with a decay rate of 30 days. **D**) Influence of suppression of double-resistance on optimal period. Here, we chose a deterministic realization, because the variability of the emergence of double-resistance is large in stochastic simulations. The black line indicates the standard setting for scenario ii (single-resistance present among incoming patients), the grey line indicates a setting where the costs of double-resistance are 50% and it is therefore not competitive.
(PDF)

Figure S8 **Considered genotypes and frequency with which infected patients become non-susceptible.** The grey, red and blue boxed indicate resistance gene loci on the bacterial chromosome (black circle). Grey boxes with a ‘+’ represent wild-type alleles, red boxes with ‘a’ and blue boxes with ‘b’ represent alleles that confer resistance to drug ‘A’ or ‘B’, respectively. Transitions between genotypes are represented by arrows and the respective mutation rate for each transition is indicated by ‘μ_x_’.
(PDF)

Figure S9 **Treatment frequencies for standard parameter setting for stochastic realizations of scenario ii (single-resistance, but not double-resistance pre-exists).** Red lines indicate patients being treated with drug A, blue lines B-treated patients. Only the last 1.5 years are shown to illustrate long-term dynamics. For each strategy, the average of 500 runs is shown.
(PDF)

Table S1 **PRISMA checklist.**
(PDF)

Table S2
**Overview of study characteristics and extracted data.** A) Study characteristics. Abbreviations: pip/taz = piperacillin/tazobactam, imi/cil = imimpinem/cilastin, tic/clav = ticarcillin/clavulanic acid. B) Extracted data. * 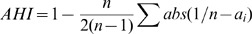 with n = number of employed antibiotics and a_i_ = usage of antibiotic a/total antibiotic usage.(PDF)

Table S3 **Results of univariate meta-analysis.**
(PDF)

Table S4 **Results of meta-analyses under inclusion of acquired instead of the total isolates in** [50], [52] **.** All other used data are the same as in figure 1 in the main text.
(PDF)

Table S5 **Results of meta-analyses under inclusion of the contemporary instead of the historic control arm in** [45] **.** All other used data are the same as in figure 1 in the main text.
(PDF)

Table S6 **Overview over parameters.** **A**) Ward characteristics [73]. * The average length of stay is 8 days in Switzerland (http://www.obsandaten.ch/indikatoren/5_4_1/2005/d/541.pdf, data from 2005) and 5 days in the US (http://www.cdc.gov/mmwr/preview/mmwrhtml/mm5427a6.htm). ** Pennsylvania Health Care Cost Containment Council. Hospital-acquired Infections in Pennsylvania 2005, 2006 & 2007. Available: http://www.phc4.org/reports/hai/(2011). ***χ was chosen arbitrarily. However, β_E_ is adjusted such that R_0_ remains constant for both direct and environmental transmission or any mixture of these two transmission modes. **B**) Composition of incoming patients [74]. **C**) Disease biology [1] [75]–[77] [78], [79] [80], [81]. * “Colonization pressure”, i.e. the frequency of both asymptomatic and symptomatic carriers in a hospital ward has been shown to be a major risk factor for the acquisition of a nosocomial pathogen [82]. It also has been shown for *Clostridium difficile*, that environmental contamination occurred for both symptomatic and asymptomatic infections [83]. Nevertheless, it is conceivable that e.g. in symptomatically infected patients with diarrhea infectivity is much higher than in asymptomatic patients. However, since the connection between carriage and infection is established and data on potential differences in infectivity between symptomatic and asymptomatic patients are scarce, we chose not to distinguish between these two classes. **D**) Treatment characteristics.
(PDF)

Text S1 **Detailed description of meta-analysis (method, extracted data and sensitivity analysis) and sensitivity analysis of theoretical model.**
(DOCX)

## References

[1] World Health Organisation (2005) Global patient safety challenge: clean care is safer care. In: World Alliance for Patient Safety, editor. Geneva.

[2] Livermore DM (2003) Bacterial resistance: origins, epidemiology, and impact. Clin Infect Dis 36: S11–23. 12516026 10.1086/344654

[3] Lambert ML, Suetens C, Savey A, Palomar M, Hiesmayr M, et al (2011) Clinical outcomes of health-care-associated infections and antimicrobial resistance in patients admitted to European intensive-care units: a cohort study. Lancet Infect Dis 11: 30–38. 21126917 10.1016/S1473-3099(10)70258-9

[4] Levin BR, Bonten MJ (2004) Cycling antibiotics may not be good for your health. Proc Natl Acad Sci U S A 101: 13101–13102. 15340145 10.1073/pnas.0404970101 PMC516530

[5] Bergstrom CT, Lo M, Lipsitch M (2004) Ecological theory suggests that antimicrobial cycling will not reduce antimicrobial resistance in hospitals. Proc Natl Acad Sci U S A 101: 13285–13290. 15308772 10.1073/pnas.0402298101 PMC516561

[6] Bonhoeffer S, Lipsitch M, Levin BR (1997) Evaluating treatment protocols to prevent antibiotic resistance. Proc Natl Acad Sci U S A 94: 12106–12111. 9342370 10.1073/pnas.94.22.12106 PMC23718

[7] Masterton RG (2005) Antibiotic cycling: more than it might seem? J Antimicrob Chemother 55: 1–5. 15574474 10.1093/jac/dkh506

[8] Masterton RG (2010) Antibiotic heterogeneity. Int J Antimicrob Agents 36 Suppl 3: S15–18. 21129627 10.1016/S0924-8579(10)70005-4

[9] Brown EM, Nathwani D (2005) Antibiotic cycling or rotation: a systematic review of the evidence of efficacy. J Antimicrob Chemother 55: 6–9. 15531594 10.1093/jac/dkh482

[10] Bal AM, Kumar A, Gould IM (2010) Antibiotic heterogeneity: from concept to practice. Ann N Y Acad Sci 1213: 81–91. 21175677 10.1111/j.1749-6632.2010.05867.x

[11] Nijssen S, Bootsma M, Bonten M (2006) Potential confounding in evaluating infection-control interventions in hospital settings: changing antibiotic prescription. Clin Infect Dis 43: 616–623. 16886156 10.1086/506438

[12] de Kraker ME, Davey PG, Grundmann H (2011) Mortality and hospital stay associated with resistant Staphylococcus aureus and Escherichia coli bacteremia: estimating the burden of antibiotic resistance in Europe. PLoS Med 8: e1001104. 22022233 10.1371/journal.pmed.1001104 PMC3191157

[13] Paul M, Kariv G, Goldberg E, Raskin M, Shaked H, et al (2010) Importance of appropriate empirical antibiotic therapy for methicillin-resistant Staphylococcus aureus bacteraemia. J Antimicrob Chemother 65: 2658–2665. 20947620 10.1093/jac/dkq373

[14] Vincent JL (2003) Nosocomial infections in adult intensive-care units. Lancet 361: 2068–2077. 12814731 10.1016/S0140-6736(03)13644-6

[15] Damas P, Canivet JL, Ledoux D, Monchi M, Melin P, et al (2006) Selection of resistance during sequential use of preferential antibiotic classes. Intensive Care Med 32: 67–74. 16308683 10.1007/s00134-005-2805-z

[16] Dominguez EA, Smith TL, Reed E, et al (2000) A pilot study of antibiotic cycling in a haematology-oncology unit. Control and Hospital Epidemiology 21: S4–S8. 10.1086/503166 10654628

[17] Evans HL, Milburn ML, Hughes MG, Smith RL, Chong TW, et al (2005) Nature of gram-negative rod antibiotic resistance during antibiotic rotation. Surg Infect (Larchmt) 6: 223–231. 16128629 10.1089/sur.2005.6.223

[18] Francetic I, Kalenic S, Huic M, Mercep I, Makar-Ausperger K, et al (2008) Impact of aminoglycoside cycling in six tertiary intensive care units: prospective longitudinal interventional study. Croat Med J 49: 207–214. 18461676 10.3325/cmj.2008.2.207 PMC2359892

[19] Gerding DNL, T A (1985) Aminoglycoside resistance in gram-negative bacilli during increased amikacin use: comparison of experience in fourteen United States hospitals with experience in the Minneapolis Veterans Administration Medical Center. American Journal of Medicine 79, Suppl. 1A: 1–7. 10.1016/0002-9343(85)90184-6 4025364

[20] Hughes MG, Evans HL, Chong TW, et al (2004) Effect of an intensive care unit rotating empiric antibiotic schedule on the development of hospital-acquired infections on the non-intensive care unit ward. Critical Care Medicine 32: 53–60. 14707559 10.1097/01.CCM.0000104463.55423.EF

[21] Kollef MH, Vlasnik J, Sharpless L, et al (1997) Scheduled rotation of antibiotic classes. A strategy to decrease the incidence of ventilator-associated pneumonia due to antibiotic-resistant gramnegative bacteria. American Journal of Respiratory Critical Care Medicine 156: 1040–1048. 9351601 10.1164/ajrccm.156.4.9701046

[22] Kollef MH, Ward S, Sherman G, et al (2000) Inadequate treatment of nosocomial infections is associated with certain empiric antibiotic choices. Critical Care Medicine 28: 3456–3464. 11057801 10.1097/00003246-200010000-00014

[23] Martinez JA, Nicolás JM, Marco F, et al (2005) Comparison of antimicrobial cycling and mixing in two medical intensive care units. Critical Care Medicine 34: 329–336. 10.1097/01.ccm.0000195010.63855.45 16424711

[24] Martinez JA, Delgado E, Marti S, Marco F, Vila J, et al (2009) Influence of antipseudomonal agents on Pseudomonas aeruginosa colonization and acquisition of resistance in critically ill medical patients. Intensive Care Med 35: 439–447. 18936910 10.1007/s00134-008-1326-y

[25] Puzniak LA, Mayfield J, Leet T, et al (2001) Acquisition of vancomycin-resistant enterococci during scheduled antimicrobial rotation in an intensive care unit. Clinical Infectious Diseases 33: 151–157. 11418873 10.1086/321807

[26] Raineri E, Crema L, Dal Zoppo S, et al (2010) Rotation of antimicrobial therapy in the intensive care unit: impact on incidence of ventilator-associated pneumonia caused by antibiotic-resistant Gram-negative bacteria. European Journal of Clinical Microbiology and Infectious Diseases 29: 1015–1024. 20524138 10.1007/s10096-010-0964-5

[27] Raymond DP, Pelletier SJ, Crabtree TD, et al (2001) Impact of a rotating empiric antibiotic schedule on infectious mortality in an intensive care unit. Critical Care Medicine 29: 1101–1108. 11395583 10.1097/00003246-200106000-00001

[28] Sandiumenge A, Diaz E, Rodriguez A, et al (2006) Impact of diversity of antibiotic use on development of antimicrobial resistance. Journal of Antimicrobial Chemotherapy 57: 1197–1204. 16565158 10.1093/jac/dkl097

[29] Sandiumenge A, Lisboa T, Gomez F, Hernandez P, Canadell L, et al (2011) Effect of antibiotic diversity on ventilator-associated pneumonia caused by ESKAPE Organisms. Chest 140: 643–651. 21659436 10.1378/chest.11-0462

[30] Takesue Y, Nakajima K, Ichiki K, et al (2010) Impact of a hospital-wide programme of heterogeneous antibiotic use on the development of antibiotic-resistant Gram-negative bacteria. Journal of Hospital Infection 75: 28–32. 20346536 10.1016/j.jhin.2009.11.022

[31] Takesue Y, Ohge H, Sakashita M, et al (2006) Effect of antibiotic heterogeneity on the development of infections with antibiotic-resistant gram-negative organisms in a nonintensive care unit surgical ward. World Journal of Surgery 30: 1269–1276. 16705389 10.1007/s00268-005-0781-7

[32] Young EJ, Sewell CM, Koza MA, et al (1985) Antibiotic resistance patterns during aminoglycoside restriction. American Journal of Medical Sciences 290: 223–227. 10.1097/00000441-198512000-00001 3936358

[33] Barie PS, Hydo LJ, Shou J, Larone DH, Eachempati SR (2005) Influence of antibiotic therapy on mortality of critical surgical illness caused or complicated by infection. Surg Infect (Larchmt) 6: 41–54. 15865550 10.1089/sur.2005.6.41

[34] Bradley SJ, Wilson AL, Allen MC, et al (1999) The control of hyperendemic glycopeptide-resistant Enterococcus spp. on a haematology unit by changing antibiotic usage. Journal of Antimicrobial Chemotherapy 43: 261–266. 11252332 10.1093/jac/43.2.261

[35] Craig M, Cumpston AD, Hobbs GR, et al (2007) The clinical impact of antibacterial prophylaxis and cycling antibiotics for febrile neutropenia in a hematological malignancy and transplantation unit. Bone Marrow Transplant 39: 477–482. 17322937 10.1038/sj.bmt.1705591

[36] Dortch MJ, Fleming SB, Kauffmann RM, Dossett LA, Talbot TR, et al (2011) Infection reduction strategies including antibiotic stewardship protocols in surgical and trauma intensive care units are associated with reduced resistant gram-negative healthcare-associated infections. Surg Infect (Larchmt) 12: 15–25. 21091186 10.1089/sur.2009.059

[37] Slain D, Sarwari AR, Petros KO, McKnight RL, Sager RB, et al (2011) Impact of a Multimodal Antimicrobial Stewardship Program on Pseudomonas aeruginosa Susceptibility and Antimicrobial Use in the Intensive Care Unit Setting. Crit Care Res Pract 2011: 416426. 21687626 10.1155/2011/416426 PMC3113284

[38] Warren DK, Hill HA, Merz LR, et al (2004) Cycling empirical antimicrobial agents to prevent emergence of antimicrobial-resistant Gram-negative bacteria among intensive care unit patients. Critical Care Medicine 32: 2450–2456. 15599150 10.1097/01.ccm.0000147685.79487.28

[39] Merz LR, Warren DK, Kollef MH, et al (2006) The impact of an antibiotic cycling program on empirical therapy for gram-negative infections. Chest 130: 1672–1678. 17166981 10.1378/chest.130.6.1672

[40] Van Loon H, Vriens M, Fluit A, et al (2005) Antibiotic rotation and development of Gram-negative antibiotic resistance. American Journal of Respiratory Critical Care Medicine 171: 480–487. 15516540 10.1164/rccm.200401-070OC

[41] Moss WJ, Beers MC, Johnson E, et al (2002) Pilot study of antibiotic cycling in a pediatric intensive care unit. Critical Care Medicine 30: 1877–1882. 12163809 10.1097/00003246-200208000-00034

[42] Pakyz ALBMF (2009) Rates of Stenotrophomonas maltophilia colonization and infection in relation to antibiotic cycling protocols. Epidemiology and Infection 137: 1679. 19874637 10.1017/S0950268809002830

[43] Hashino S, Morita L, Kanamori H, Takahata M, Onozawa M, et al (2012) Clinical impact of cycling the administration of antibiotics for febrile neutropenia in Japanese patients with hematological malignancy. Eur J Clin Microbiol Infect Dis 31: 173–178. 21594713 10.1007/s10096-011-1290-2

[44] Bruno-Murtha LA, Brusch J, Bor D, et al (2005) A pilot study of antibiotic cycling in the community hospital setting. Infection Control Hospital Epidemiology 26: 81–87. 15693413 10.1086/502491

[45] Bennett KM, Scarborough JE, Sharpe M, et al (2007) Implementation of antibiotic rotation protocol improves antibiotic susceptibility profile in a surgical intensive care unit. Journal of Trauma 63: 307–311. 17693828 10.1097/TA.0b013e318120595e

[46] Gerding DN, Larson TA, Hughes RA, et al (1991) Aminoglycoside resistance and aminoglycoside usage: ten years of experience in one hospital. Antimicrobial Agents and Chemotherapy 35. 10.1128/aac.35.7.1284 PMC245159 1929283

[47] Gruson D, Hilbert G, Vargas F, et al (2000) Rotation and restricted use of antibiotics in a medical intensive care unit: impact on the incidence of ventilator-associated pneumonia caused by antibioticresistant Gram-negative bacteria. American Journal of Respiratory Critical Care Medicine 162: 837–843. 10988092 10.1164/ajrccm.162.3.9905050

[48] Gruson D, Hilbert G, Vargas F, et al (2003) Strategy of antibiotic rotation: long term effect on incidence and susceptibilities of Gram-negative bacilli responsible for ventilator-associated pneumonia.∧. Critical Care Medicine 31: 1908–1914. 12847382 10.1097/01.CCM.0000069729.06687.DE

[49] Cadena J, Taboada CA, Burgess DS, Ma JZ, Lewis JS 2nd, et al (2007) Antibiotic cycling to decrease bacterial antibiotic resistance: a 5-year experience on a bone marrow transplant unit. Bone Marrow Transplant 40: 151–155. 17530005 10.1038/sj.bmt.1705704

[50] Hedrick TL, Schulman AS, McElearney ST, et al (2008) Outbreak of resistant Pseudomonas aeruginosa infections during a quarterly cycling antibiotic regimen. Surgical Infections 9: 139–152. 18426346 10.1089/sur.2006.102

[51] Kheder SI, Eltayeb I, Shaddad SA, AlKhedir I (2012) Optimizing Antimicrobial Drug Use in Surgery: An Intervention Strategy in A Sudanese Hospital to Combat The Emergence of Bacterial Resistant. Sudan Journal of Medical Sciences 6.

[52] Nijssen S, Fluit A, van de Vijver D, et al (2010) Effects of reducing beta-lactam antibiotic pressure on intestinal colonization of antibiotic-resistant gram-negative bacteria. Intensive Care Medicine 36: 512–519. 19921150 10.1007/s00134-009-1714-y PMC2820219

[53] Toltzis P, Dul M, Hoyen C, et al (2002) The effect of antibiotic rotation on colonization with antibiotic-resistant bacilli in a neonatal intensive care unit. Peaditrics 110: 707–711. 10.1542/peds.110.4.707 12359783

[54] Smith RL, Evans HL, Chong TW, et al (2008) Reduction in rates of methicillin-resistant Staphylococcus aureus infection after introduction of quarterly linezolidvancomycin cycling in a surgical intensive care unit. Surgical Infections 9: 423–431. 18759679 10.1089/sur.2007.024 PMC2996816

[55] Ginn AN, Wiklendt AM, Gidding HF, George N, O'Driscoll JS, et al (2012) The ecology of antibiotic use in the ICU: homogeneous prescribing of cefepime but not tazocin selects for antibiotic resistant infection. PLoS One 7: e38719. 22761698 10.1371/journal.pone.0038719 PMC3382621

[56] Cumpston A, Craig M, Hamadani M, Abraham J, Hobbs GR, et al (2013) Extended follow-up of an antibiotic cycling program for the management of febrile neutropenia in a hematologic malignancy and hematopoietic cell transplantation unit. Transpl Infect Dis 15: 142–149. 23279656 10.1111/tid.12035

[57] Kontopidou FV, Antoniadou A, Tsirigotis P, Venetis E, Polemis M, et al (2013) The impact of an antimicrobial cycling strategy for febrile neutropenia in a haematology unit. J Chemother 25: 279–285. 24070135 10.1179/1973947813Y.0000000077

[58] Sarraf-Yazdi S, Sharpe M, Bennett KM, Dotson TL, Anderson DJ, et al (2012) A 9-Year retrospective review of antibiotic cycling in a surgical intensive care unit. J Surg Res 176: e73–78. 22445457 10.1016/j.jss.2011.12.014 PMC3721312

[59] Chong Y, Shimoda S, Yakushiji H, Ito Y, Miyamoto T, et al (2013) Antibiotic rotation for febrile neutropenic patients with hematological malignancies: clinical significance of antibiotic heterogeneity. PLoS One 8: e54190. 23372683 10.1371/journal.pone.0054190 PMC3553165

[60] Merz LR, Warren DK, Kollef MH, Fraser VJ (2004) Effects on antibiotic cycling programon antibiotic prescribing practices in an intensive care unit. Antimicrobial Agents and Chemotherapy 48: 2861–2865. 15273092 10.1128/AAC.48.8.2861-2865.2004 PMC478533

[61] Beardmore RE, Pena-Miller R (2010) Antibiotic cycling versus mixing: the difficulty of using mathematical models to definitively quantify their relative merits. Math Biosci Eng 7: 923–933. 21077716 10.3934/mbe.2010.7.923

[62] Chow K, Wang X, Curtiss R 3rd, Castillo-Chavez C (2011) Evaluating the efficacy of antimicrobial cycling programmes and patient isolation on dual resistance in hospitals. J Biol Dyn 5: 27–43. 22877228 10.1080/17513758.2010.488300

[63] Martínez JA, Nicolás JM, Marco F, et al (2005) Comparison of antimicrobial cycling and mixing in two medical intensive care units. Critical Care Medicine 34: 329–336. 10.1097/01.ccm.0000195010.63855.45 16424711

[64] Kouyos RD, Abel Zur Wiesch P, Bonhoeffer S (2011) Informed switching strongly decreases the prevalence of antibiotic resistance in hospital wards. PLoS Comput Biol 7: e1001094. 21390265 10.1371/journal.pcbi.1001094 PMC3048378

[65] Viechtbauer W (2010) Conducting meta-analyses in R with the metafor package. Journal of

[66] Berkey CS, Hoaglin DC, Antczak-Bouckoms A, Mosteller F, Colditz GA (1998) Meta-analysis of multiple outcomes by regression with random effects. Stat Med 17: 2537–2550. 9839346 10.1002/(sici)1097-0258(19981130)17:22<2537::aid-sim953>3.0.co;2-c

[67] Riley RD, Thompson JR (2008) An alternative model for bivariate random-effects meta-analysis when the within-study correlations are unknown. Biostatistics 9: 172–186. 17626226 10.1093/biostatistics/kxm023

[68] Riley RD (2009) Multivariate meta-analysis: The effect of ignoring within-study correlation. Journal of the Royal Statistical Society, Series A 178: 789–811.

[69] Higgins JP, Thompson SG, Deeks JJ, Altman DG (2003) Measuring inconsistency in meta-analyses. BMJ 327: 557–560. 12958120 10.1136/bmj.327.7414.557 PMC192859

[70] Egger M, Davey Smith G, Schneider M, Minder C (1997) Bias in meta-analysis detected by a simple, graphical test. BMJ 315: 629–634. 9310563 10.1136/bmj.315.7109.629 PMC2127453

[71] Begg CB, Mazumdar M (1994) Operating characteristics of a rank correlation test for publication bias. Biometrics 50: 1088–1101. 7786990

[72] Kouyos RD, Abel Zur Wiesch P, Bonhoeffer S (2011) On being the right size: the impact of population size and stochastic effects on the evolution of drug resistance in hospitals and the community. PLoS Pathog 7: e1001334. 21533212 10.1371/journal.ppat.1001334 PMC3077359

[73] Neely AN, Maley MP (2000) Survival of enterococci and staphylococci on hospital fabrics and plastic. J Clin Microbiol 38: 724–726. 10655374 10.1128/jcm.38.2.724-726.2000 PMC86187

[74] European Center for Disease Prevention and Control (2008) Annual Epidemiological Report on Communicable Diseases in Europe 2008. In: Control ECfDPa, editor. Stockholm.

[75] Luciani F, Sisson SA, Jiang H, Francis AR, Tanaka MM (2009) The epidemiological fitness cost of drug resistance in Mycobacterium tuberculosis. Proc Natl Acad Sci U S A 106: 14711–14715. 19706556 10.1073/pnas.0902437106 PMC2732896

[76] Trindade S, Sousa A, Xavier KB, Dionisio F, Ferreira MG, et al (2009) Positive epistasis drives the acquisition of multidrug resistance. PLoS Genet 5: e1000578. 19629166 10.1371/journal.pgen.1000578 PMC2706973

[77] Johnsen PJ, Townsend JP, Bohn T, Simonsen GS, Sundsfjord A, et al (2011) Retrospective evidence for a biological cost of vancomycin resistance determinants in the absence of glycopeptide selective pressures. J Antimicrob Chemother 66: 608–610. 21217128 10.1093/jac/dkq512 PMC3037156

[78] Dennesen PJ, van der Ven AJ, Kessels AG, Ramsay G, Bonten MJ (2001) Resolution of infectious parameters after antimicrobial therapy in patients with ventilator-associated pneumonia. Am J Respir Crit Care Med 163: 1371–1375. 11371403 10.1164/ajrccm.163.6.2007020

[79] Ottiger C, Schaer G, Huber AR (2007) Time-course of quantitative urinary leukocytes and bacteria counts during antibiotic therapy in women with symptoms of urinary tract infection. Clin Chim Acta 379: 36–41. 17229419 10.1016/j.cca.2006.11.023

[80] Grundmann H, Hellriegel B (2006) Mathematical modelling: a tool for hospital infection control. Lancet Infect Dis 6: 39–45. 16377533 10.1016/S1473-3099(05)70325-X

[81] Bootsma MC, Wassenberg MW, Trapman P, Bonten MJ (2011) The nosocomial transmission rate of animal-associated ST398 meticillin-resistant Staphylococcus aureus. J R Soc Interface 8: 578–584. 20861037 10.1098/rsif.2010.0349 PMC3061118

[82] Bonten MJ, Hayden MK, Nathan C, Rice TW, Weinstein RA (1998) Stability of vancomycin-resistant enterococcal genotypes isolated from long-term-colonized patients. J Infect Dis 177: 378–382. 9466524 10.1086/514196

[83] McFarland LV, Mulligan ME, Kwok RY, Stamm WE (1989) Nosocomial acquisition of Clostridium difficile infection. N Engl J Med 320: 204–210. 2911306 10.1056/NEJM198901263200402

