# Supplementary figures and images for: Cycling Empirical Antibiotic Therapy in Hospitals: Meta-Analysis and Models
Source: PLoS Pathog. 2014 Jun 26;10(6):e1004225. doi: 10.1371/journal.ppat.1004225 (PMC4072793; doi:10.1371/journal.ppat.1004225)

A

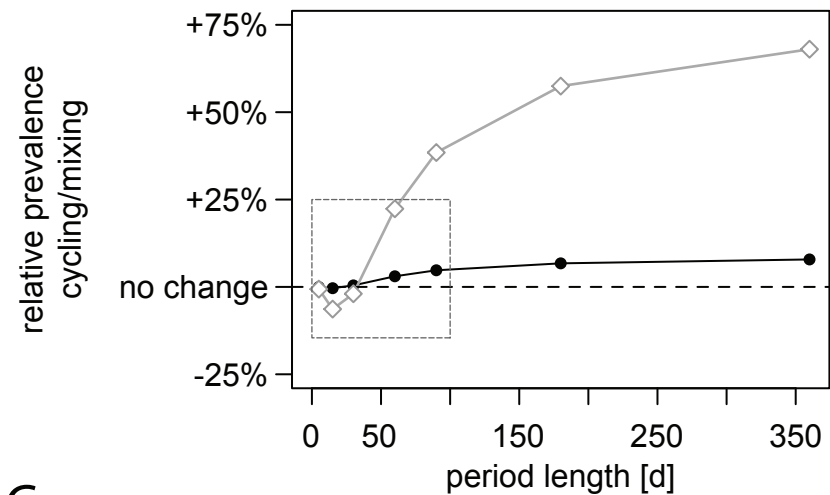

B

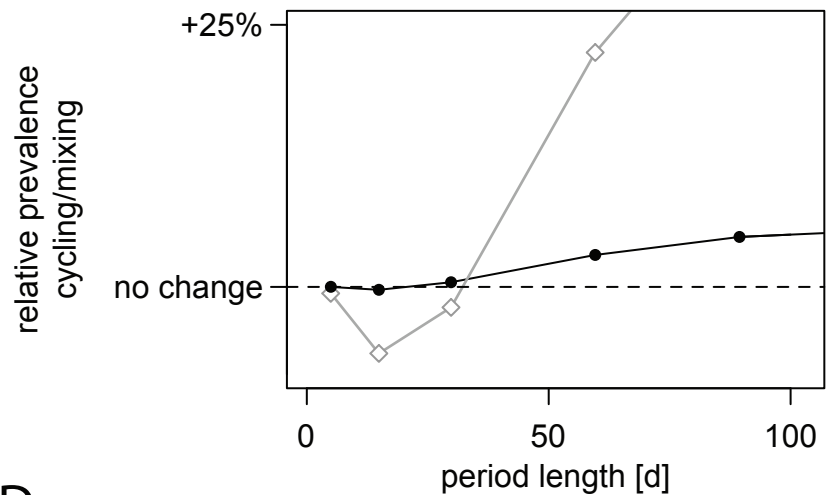

C

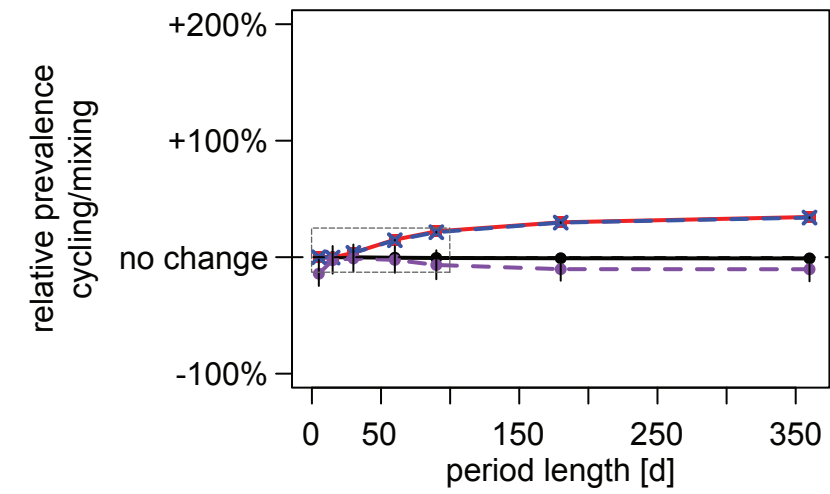

D

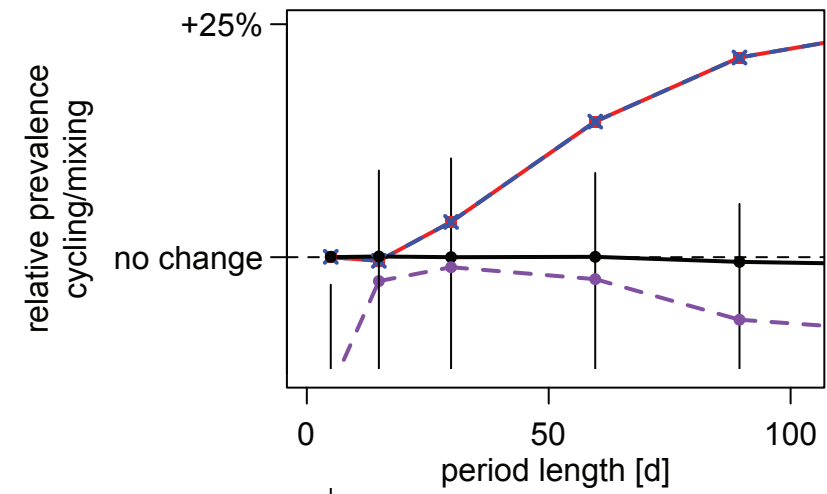

Supplement: Figure S2 — Influence of period length on “adjustable cycling” success and genotype composition. Results of stochastic simulations with parameters for scenario ii (single-resistance present among incoming patients) and direct transmission. A) Relative change of inappropriately treated patients (open grey diamonds) and symptomatically infected patients (solid black circles) as compared to “adjustable mixing”. B) same as A with higher resolution. C) Relative change of genotype composition depending on the period length as compared to “adjustable mixing”: black indicates wild-type, red resistance to A, blue resistance to B, and dotted purple resistance to both drugs. D) same as C) with higher resolution. The 95% CI, as determined by bootstrapping, is given as error bars. Please note that the error bars for all measures except the prevalence of double-resistance are smaller than the used symbols. (PDF) [file ppat.1004225.s002.pdf]

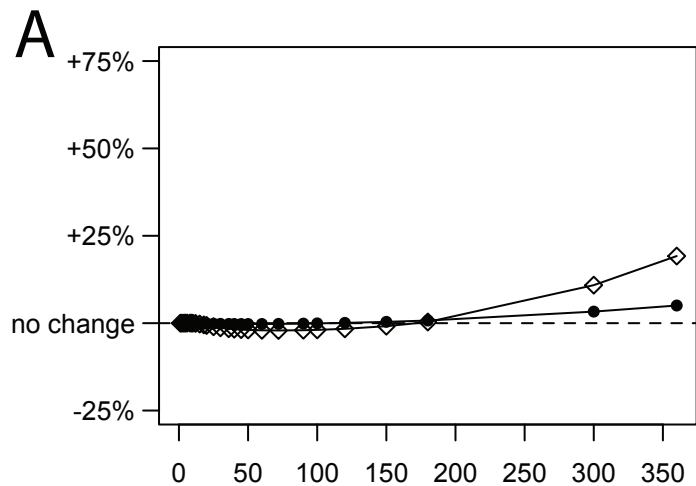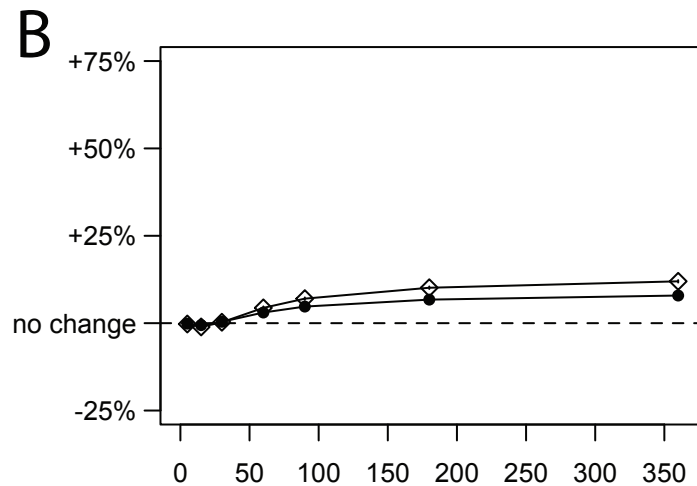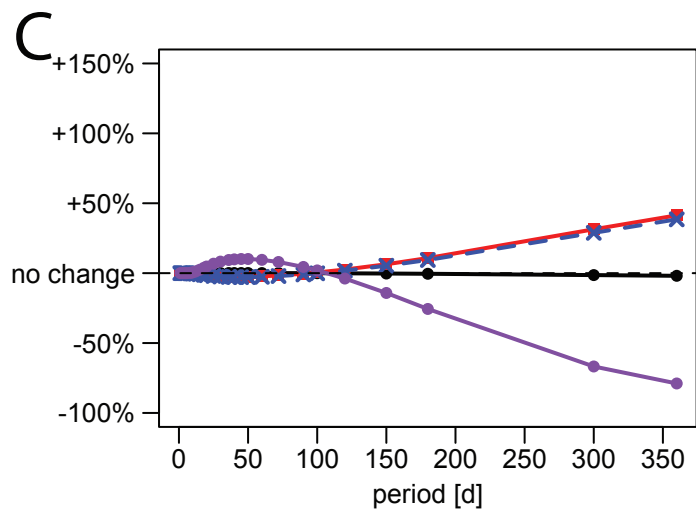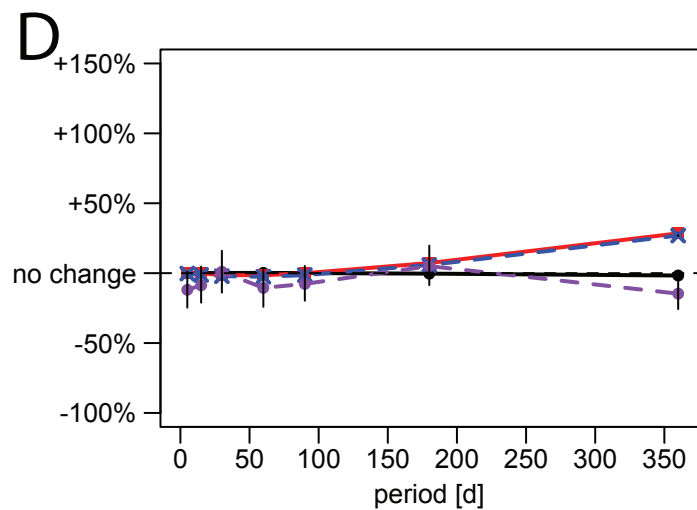

Supplement: Figure S3 — Influence of period length on cycling success and genotype composition, environmental transmission. For all simulations, parameters for scenario ii (single-resistance present among incoming patients) and environmental transmission were used. A) Relative change of inappropriately treated patients (open diamonds) and symptomatically infected patients (solid circles) as compared to mixing for a deterministic realization. B) Same as A) for a stochastic realization. C) Relative change of genotype composition depending on the period length as compared to mixing: black indicates wild-type, red resistance to A, blue resistance to B, and dotted purple resistance to both drugs. The 95% CI, as determined by bootstrapping, is given as error bars. D) Same as C) for a stochastic realization. (PDF) [file ppat.1004225.s003.pdf]

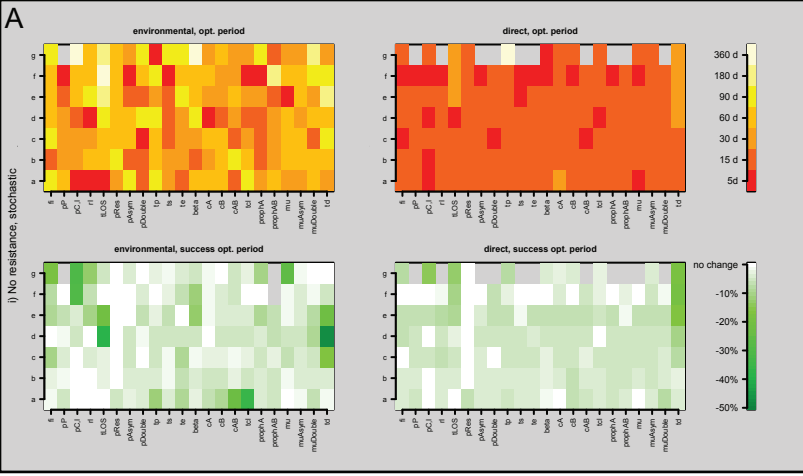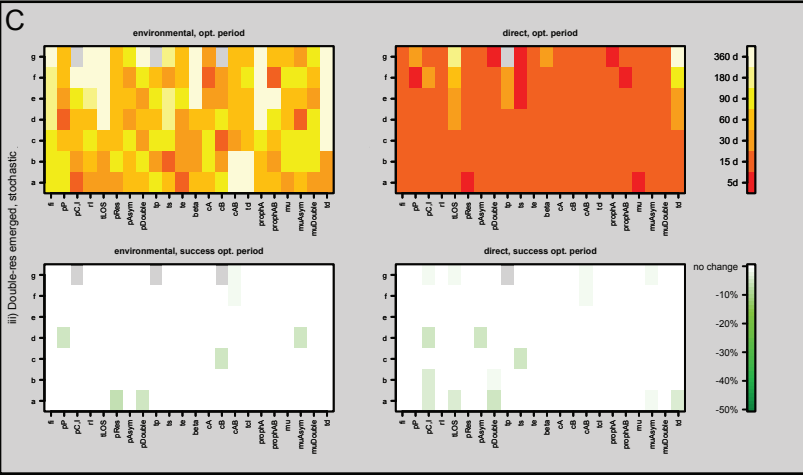

Supplement: Figure S4 — Overview over optimal cycling periods and success of these periods. A) For stochastic simulations if no resistance pre-exists in the incoming patients (scenario i). The results were obtained by averaging over 10000 simulations. Parameters (explained in table S6) were varied from a) (lowest) to g) (highest, see table S6) and the effect on the relative reduction of inappropriately treated patients as compared to mixing is indicated by green color code for an optimal period length. The optimal period was defined as the period that is most successful in reducing inappropriate therapy without leading to a higher prevalence of symptomatic infections and its length is indicated by red color code. The areas shaded in grey indicate that there is no period (within our screened range) for which cycling outperforms mixing. B) For stochastic simulations if only single-resistance pre-exists in the incoming patients (scenario ii). The results were obtained by averaging over 10000 simulations. Parameters (explained in table S6 were varied from a) (lowest) to g) (highest, see table S6 and the effect on the relative reduction of inappropriately treated patients as compared to mixing is indicated by green color code for an optimal period length. The optimal period was defined as the period that is most successful in reducing inappropriate therapy without leading to a higher prevalence of symptomatic infections and its length is indicated by red color code. The areas shaded in grey indicate that there is no period (within our screened range) for which cycling outperforms mixing. C) For stochastic simulations if both single and double-resistance pre-exist in the incoming patients (scenario iii). The results were obtained by averaging over 10000 simulations. Parameters (explained in table S6) were varied from a) (lowest) to g) (highest, see table S6 and the effect on the relative reduction of inappropriately treated patients as compared to mixing is indicated by green color code for an o [file ppat.1004225.s004.pdf]

**A****Proportion Susceptible**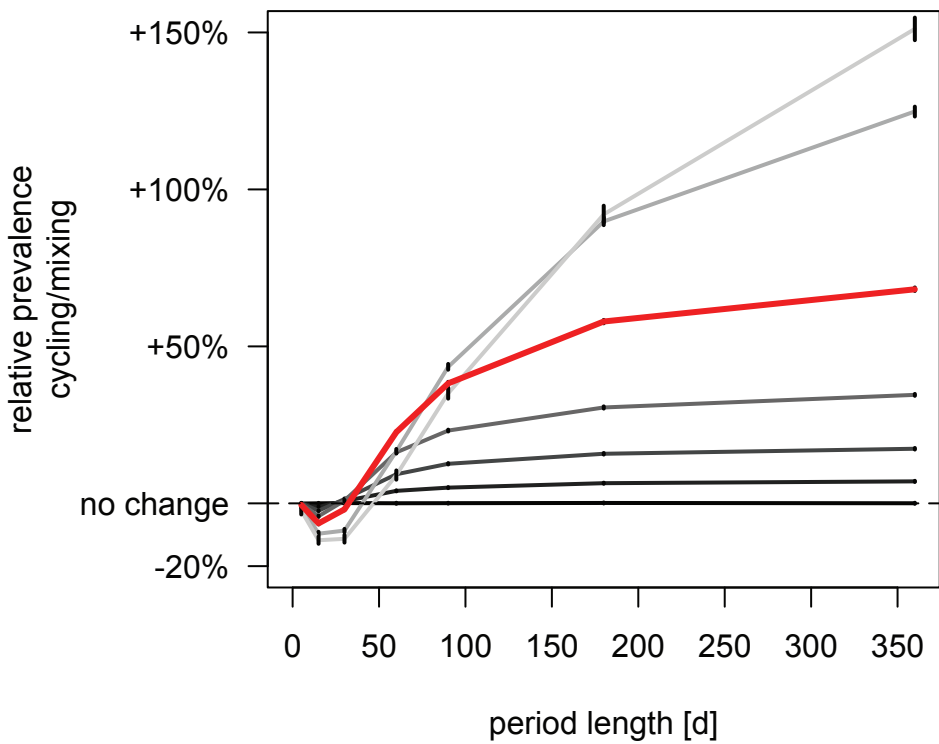**B****Environmental decay rate**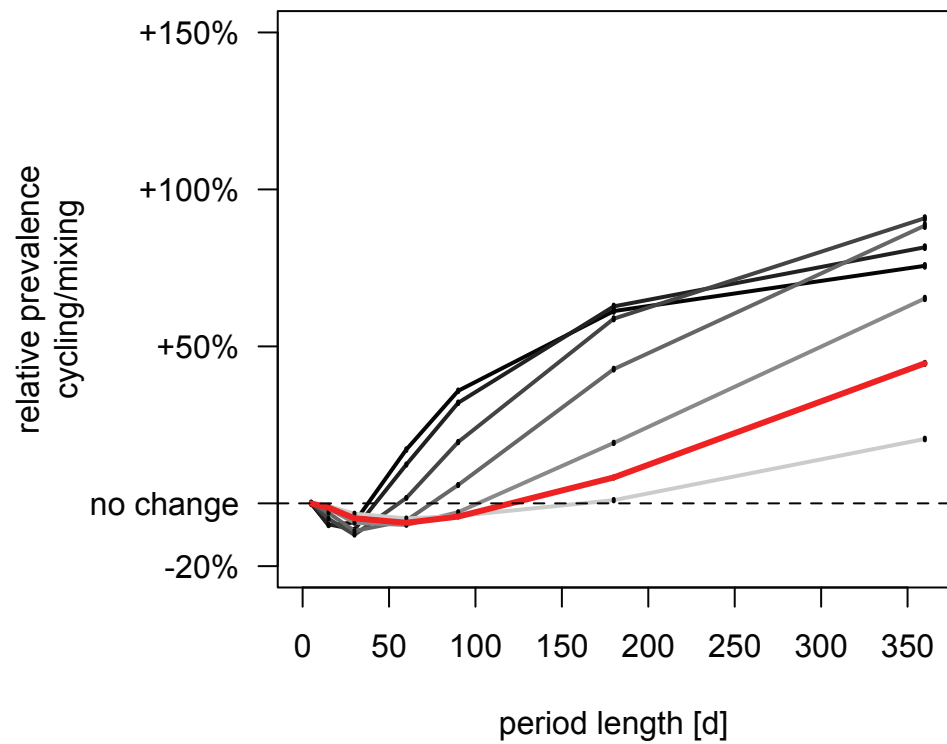

Supplement: Figure S5 — Influence of admission of susceptible patients and time between first and second transmission. The lines represent the averages of 10000 stochastic simulations; the error bars the 95% CI as determined by bootstrapping. The red line indicates the standard parameter setting. A) Shows an example of increasing the influx of susceptible patients (from 0%, given in black in 15% steps to 90%, given in light grey). Simulations for direct transmission for scenario ii (only single-resistance among incoming patients) are shown. B) Shows an example of increasing the time between the colonization of a patient and the transmission of the pathogen by increasing the environmental decay rate (from 1 day, given in black over 2, 5, 7, 10, 20 to 100 days, given in light grey). Simulations for environmental transmission for scenario ii (only single-resistance among incoming patients) are shown. The dotted black line indicates no difference in prevalence. (PDF) [file ppat.1004225.s005.pdf]

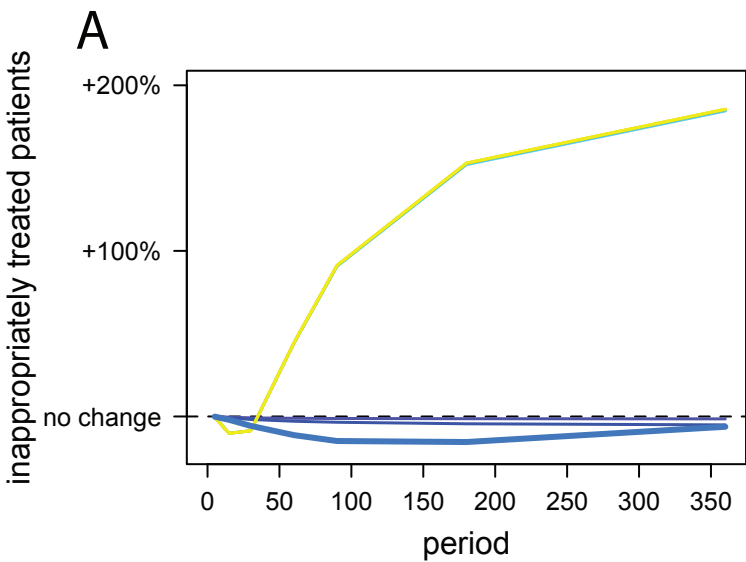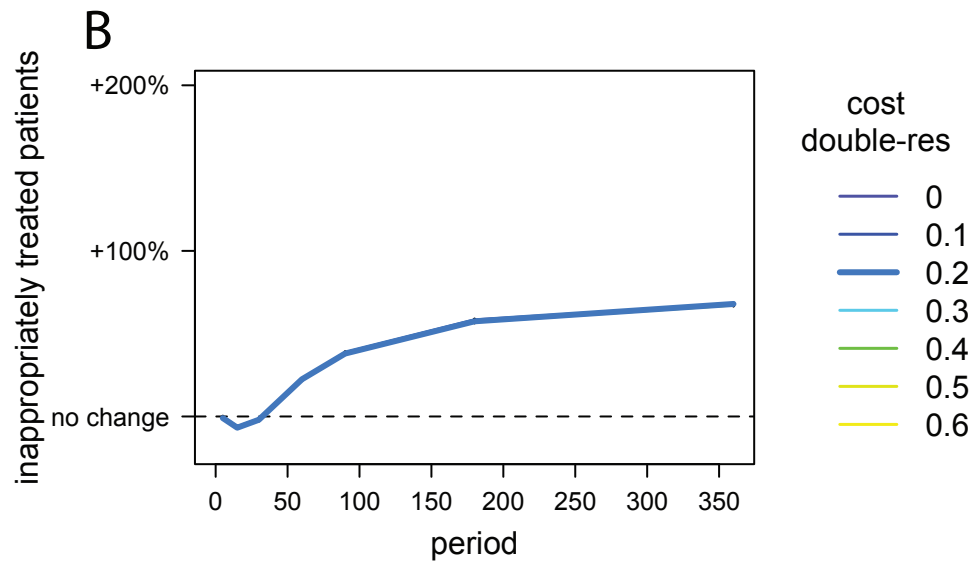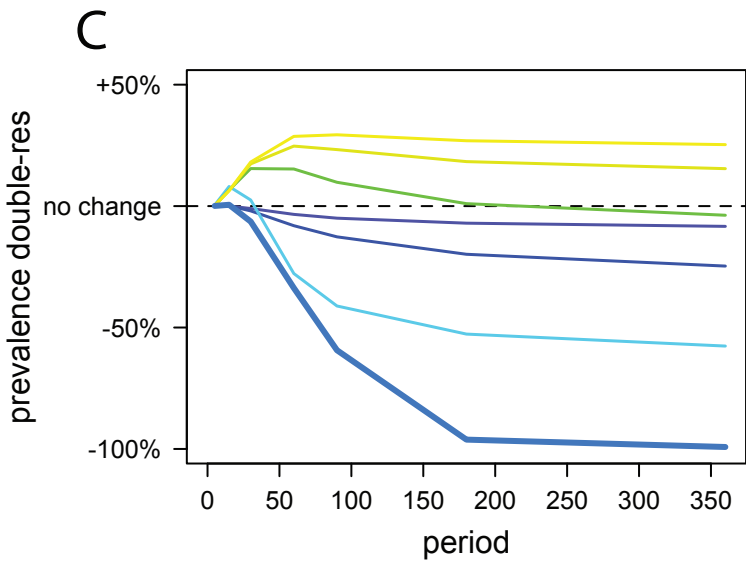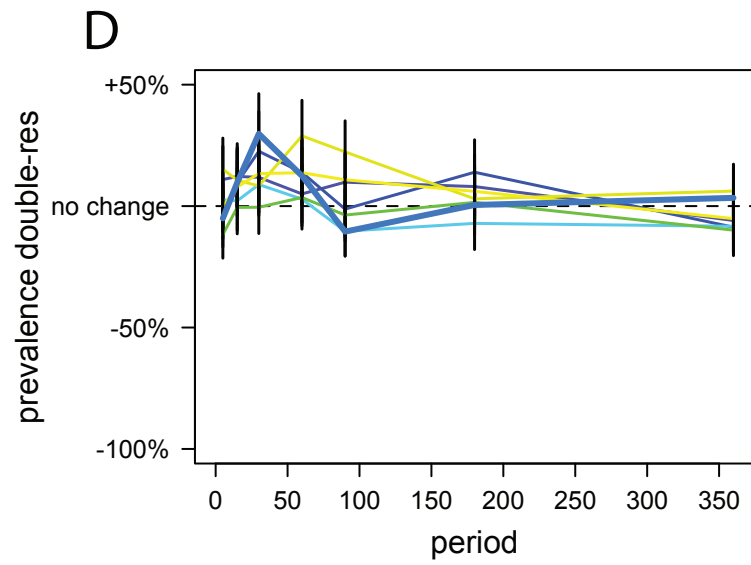

Supplement: Figure S6 — “Adjustable cycling” success depends on fitness of double-resistant strain. These graphs show how the suppression of double-resistance depends on the fitness of the double-resistant strain (color code on the right). The parameter setting corresponds to scenario ii (single-resistance among incoming patients) for direct transmission. The upper panel (A, B) gives the prevalence of inappropriately treated patients relative to mixing; A) for deterministic realizations and B) for the stochastic realizations. The 95% CI, as determined by bootstrapping, is given as error bars, however, these are smaller than the line width. The lower panel (C, D) gives the prevalence of double-resistant strains relative to mixing; C) for deterministic realizations and D) for the stochastic realizations. The 95% CI, as determined by bootstrapping, is given as error bars. (PDF) [file ppat.1004225.s006.pdf]

A

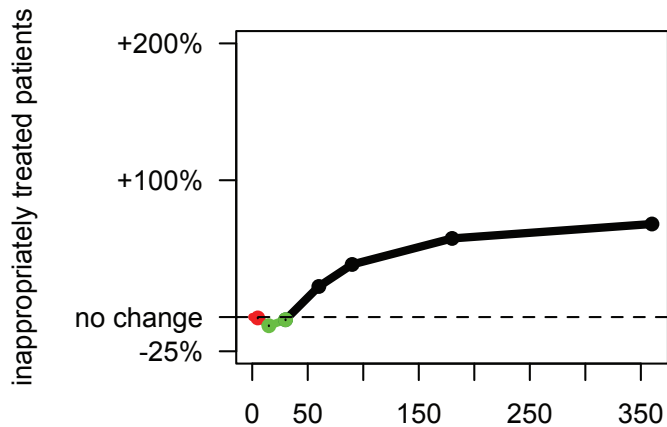

B

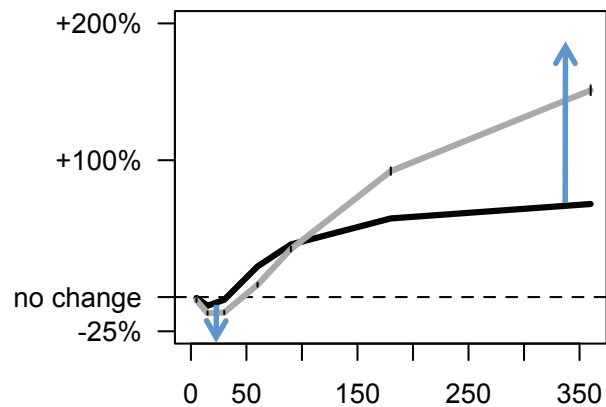

C

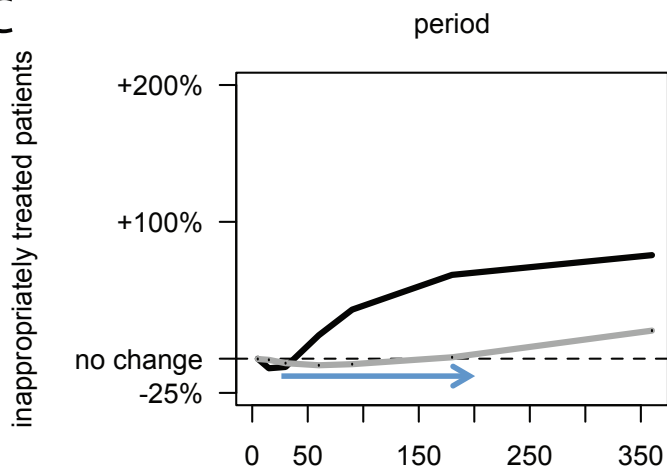

D

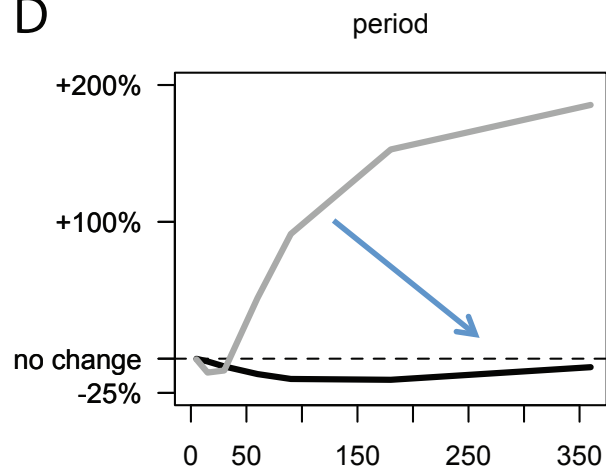

Supplement: Figure S7 — Selection pressure depending on period length and main factors influencing the optimal period. A) Classification of period lengths in three groups, on the example of a stochastic realization of the standard setting for scenario ii (single-resistance present among incoming patients). i) red indicates those periods for which there is no difference between “adjustable mixing” and “adjustable cycling”, ii) green indicates those periods, which select against single-resistance and for double-resistance, iii) black those that both increasingly select against double-resistance and increasingly allow outgrowth of single-resistant strains. B) to D) show examples of the main factors influencing the location and success of the optimal period, the arrows summarize the trends which occur by changing these factors. B) Influence of the difference between the prevalences of the single resistant strains during the on- and off-periods in “adjustable cycling”. The black line indicates the standard value as in A), the grey line indicates a setting with more incoming susceptible patients C) Influence of the turnover rate on the optimal period. The black line indicates environmental transmission with a decay rate of one day (note the similarity to the standard setting for direct transmission), the grey line indicates environmental transmission with a decay rate of 30 days. D) Influence of suppression of double-resistance on optimal period. Here, we chose a deterministic realization, because the variability of the emergence of double-resistance is large in stochastic simulations. The black line indicates the standard setting for scenario ii (single-resistance present among incoming patients), the grey line indicates a setting where the costs of double-resistance are 50% and it is therefore not competitive. (PDF) [file ppat.1004225.s007.pdf]

$\mu_a$

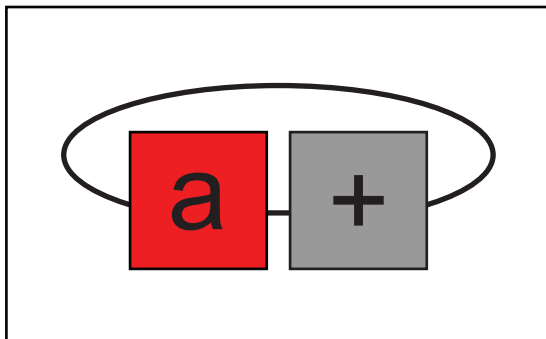

$\mu_b$

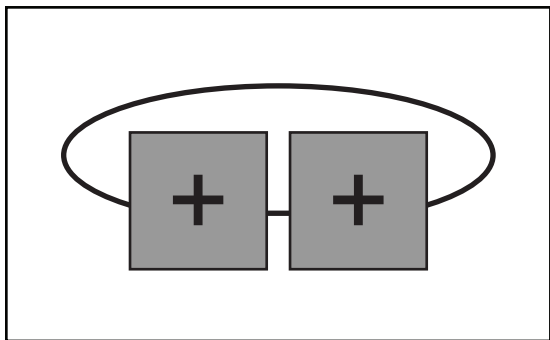

$\mu_{ab}$

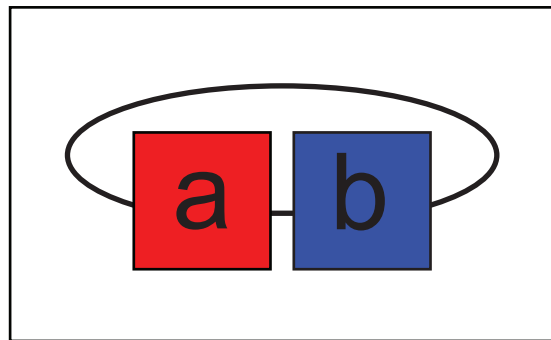

$\mu_b$

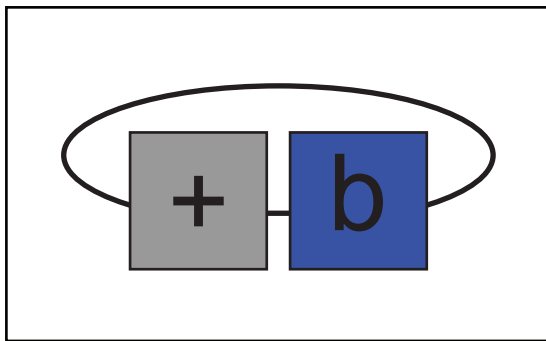

$\mu_a$

Supplement: Figure S8 — Considered genotypes and frequency with which infected patients become non-susceptible. The grey, red and blue boxed indicate resistance gene loci on the bacterial chromosome (black circle). Grey boxes with a ‘+’ represent wild-type alleles, red boxes with ‘a’ and blue boxes with ‘b’ represent alleles that confer resistance to drug ‘A’ or ‘B’, respectively. Transitions between genotypes are represented by arrows and the respective mutation rate for each transition is indicated by ‘μx’. (PDF) [file ppat.1004225.s008.pdf]

**Mixing, treatment frequency**

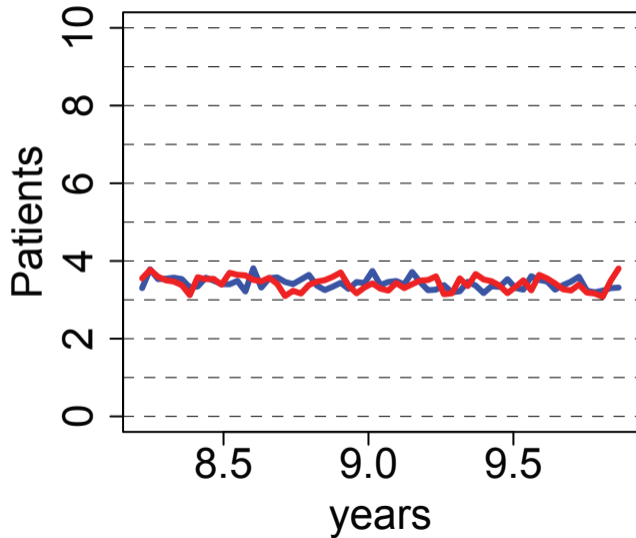

**Cycling, treatment frequency**

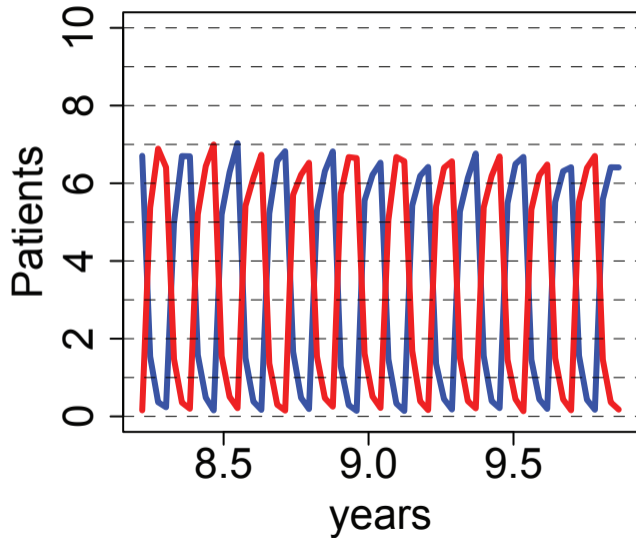

Supplement: Figure S9 — Treatment frequencies for standard parameter setting for stochastic realizations of scenario ii (single-resistance, but not double-resistance pre-exists). Red lines indicate patients being treated with drug A, blue lines B-treated patients. Only the last 1.5 years are shown to illustrate long-term dynamics. For each strategy, the average of 500 runs is shown. (PDF) [file ppat.1004225.s009.pdf]
