# Supplementary material for: Cycling Empirical Antibiotic Therapy in Hospitals: Meta-Analysis and Models
Source: PLoS Pathog. 2014 Jun 26;10(6):e1004225. doi: 10.1371/journal.ppat.1004225 (PMC4072793; doi:10.1371/journal.ppat.1004225)
Supplement: Table S2 — Overview of study characteristics and extracted data. A) Study characteristics. Abbreviations: pip/taz = piperacillin/tazobactam, imi/cil = imimpinem/cilastin, tic/clav = ticarcillin/clavulanic acid. B) Extracted data. * with n = number of employed antibiotics and ai = usage of antibiotic a/total antibiotic usage. (PDF) [file ppat.1004225.s011.pdf]

A

	Bennett et al.	Cadena et al.	Gerding et al.	Gruson et al.	Gruson et al.	Hedrick et al.	Kheder et al.	Nijssen et al.	Smith et al.	Toltzis et al.	Chong et al.
**Year**	2007	2007	1991	2000	2003	2008	2011	2010	2008	2002	2013
**Isolates**	total	total	total	total	total	acquired	total	acquired	total	total	total
**Total**	no	no	no	yes	yes	yes	yes	no	yes	yes	yes
**Gram-negative**	no	no	yes	yes	yes	yes	yes	no	yes	yes	yes
P. aeruginosa	yes	yes	yes	yes	yes	yes	yes	no	no	yes	yes
**Enterobacteriaceae**	no	yes	yes	yes	yes	yes	yes	yes	no	yes	no
E. coli	yes	no	yes	yes	yes	yes	yes	no	no	yes	yes
K. pneumoniae	yes	no	yes	yes	yes	yes	yes	no	no	yes	yes
**Gram-positive**	no	no	no	yes	yes	yes	yes	no	yes	no	yes
S. aureus	no	yes	no	yes	yes	yes	yes	no	yes	no	yes
**Enterococci**	no	no	no	no	no	yes	yes	no	yes	no	yes
**Control Period**	temporal	temporal	temporal	temporal	temporal	tem poral	temporal	temporal	temporal	spatial	temporal
**Complete repetition**	yes	yes	yes	yes	yes	no	yes	yes	yes	yes	yes
**Control for Import**	no	no	no	no	no	yes	no	yes	yes	yes	no
**Setting**	ICU	ICU	hospital	ICU	ICU	ICU	ward	ICU	ICU	ICU	ICU
**Drug 1**	pip/ taz	pip/ taz	amikacin	cefepime	cefepime	cefepime	cephalosporins	ceftriaxone	linezolid	gentamicin	pip/ taz
**Drug 2**	imi/ cil	cefepime	gentamicin	pip/ taz	pip/ taz	ciprofloxacin	co-amoxiclav	co-amoxiclav	vancomycin	pip/ taz	ciprofloxacin
**Drug 3**	ceftazidime	NA	NA	imipenem	imipenem	pip/ taz	ciprofloxacin	fluoroquinolone	NA	ceftazidime	meropenem
**Drug 4**	ciprofloxacin	NA	NA	tic/ clav	tic/ clav	imi/ cil	NA	NA	NA	NA	cefepime
**Class change**	no	no	no	no	no	no	yes	yes	yes	yes	no

B

**General**	**Bennett et al.**	**Cadena et al.**	**Gerding et al.**	**Gruson et al.**	**Gruson et al.**	**Hedrick et al.**	**Kheder et al. (GIT)**	**Kheder et al. (Urology)**	**Nijssen et al.**	**Smith et al.**	**Toltzis et al.**	**Chong et al.**
Year	2007	2007	1991	2000	2003	2008	2011	2011	2010	2008	2002	2013
# of beds	16	11	700	16	16	16	NA	NA	18	NA	19	37
Length of stay [d]	4.8	NA	NA	6.67	NA	9.83	9.65	8.81	7.5	4.27	11.3	32
**Cycling**												
Duration cycling [mo]	12	60	85	24	36	18	21	21	3	24	12	20
Cycle length [mo]	1	3	23.75	1	1	3	3.5	3.5	0.25	3	1	1
# of drugs	4	2	2	4	4	4	3	3	3	2	3	4
Adherence	NA	NA	0.81	NA	NA	0.37	0.52	0.57	NA	0.8	0.84	NA
Weighted resistance/ isolate*	0.77	NA	0.03	1.33	1.65	2.33	2.23	2.6	2.13	0.37	1.78	0.09
Total isolates/ patient day	0.016	0.016	0.013	0.02	0.011	0.093	0.005	0.012	0.019	0.02	0.023	0.0072
Resistant isolates/ patient day	0.012	NA	0	0.018	0.013	0.061	0.011	0.03	0.04	0.007	0.035	0.00065
Resistant isolates 1. antibiotic/ patient day	0.005	NA	0	0.004	0.003	0.016	0.004	0.011	0.019	NA	0.01	0
Resistant isolates following antibiotic/ patient day	0.008	NA	0.001	0.014	0.01	0.045	0.007	0.019	0.021	NA	0.025	0.00065
Single resistant isolates/ patient day	NA	NA	NA	NA	NA	NA	NA	NA	0.016	NA	NA	NA
Multiple resistant isolates/ patient day	NA	NA	NA	NA	NA	0.0119	NA	NA	0.0025	NA	NA	NA
Deaths/ patient day	NA	NA	NA	0.0052	0.0028	0.0068	NA	NA	0.0142	NA	0.0026	0.00087
**Baseline**												
Duration baseline [mo]	12	24	3	24	24	4	8	8	8	48	12	12
Defined daily doses (DDD)	NA	NA	3.4	115.9	88.2	NA	24.3	56.5	98	NA	94.1	3.35
Weighted resistance/ isolate*	1.29	NA	1.71	1.14	1.14	2.45	2.43	2.67	2.16	0.23	1.87	0.65
Total isolates/ patient day	0.018	0.015	0.015	0.029	0.029	0.099	0.008	0.007	0.023	0.022	0.019	0.0098
Resistant isolates/ patient day	0.023	NA	0.026	0.024	0.024	0.088	0.02	0.017	0.05	0.005	0.029	0.0064
Resistant isolates 1. antibiotic/ patient day	0.012	NA	0.001	0.004	0.004	0.019	0.007	0.006	0.023	NA	0.01	0
Resistant isolates following antibiotic/ patient day	0.012	NA	0.002	0.02	0.02	0.069	0.013	0.011	0.027	NA	0.019	0.0047
Single resistant isolates/ patient day	NA	NA	NA	NA	NA	NA	NA	NA	0.019	NA	NA	NA
Multiple resistant isolates/ patient day	NA	NA	NA	NA	NA	0.019	NA	NA	0.004	NA	NA	NA
Deaths/ patient day	NA	NA	NA	0.008	0.008	0.009	NA	NA	0.019	NA	0.002	0.00085
**Ratios**												
AHI cycling/ baseline *	NA	293.33	1.51	1.27	1.27	1.22	1.31	2	1.06	1.9	3.72	0.53
AHI during each cyc. period/ baseline *	NA	NA	0.85	NA	NA	1.06	NA	NA	NA	NA	1.61	NA
DDD cycling/ baseline	NA	NA	1.89	0.47	0.5	NA	2.06	2.43	0.69	NA	0.93	0.94
