# Supplementary material for: Cycling Empirical Antibiotic Therapy in Hospitals: Meta-Analysis and Models
Source: PLoS Pathog. 2014 Jun 26;10(6):e1004225. doi: 10.1371/journal.ppat.1004225 (PMC4072793; doi:10.1371/journal.ppat.1004225)
Supplement: Table S3 — Results of univariate meta-analysis. (PDF) [file ppat.1004225.s012.pdf]

	estimate	lower CI	upper CI	p-value
**Total**	-4.6170	-9.1276	-0.1064	0.0448
**Resistant**	-7.4996	-14.3173	-0.6818	0.0311
**Deaths**	-2.1604	-3.9921	-0.3287	0.0208
