# Supplementary material for: Cycling Empirical Antibiotic Therapy in Hospitals: Meta-Analysis and Models
Source: PLoS Pathog. 2014 Jun 26;10(6):e1004225. doi: 10.1371/journal.ppat.1004225 (PMC4072793; doi:10.1371/journal.ppat.1004225)
Supplement: Table S4 — Results of meta-analyses under inclusion of acquired instead of the total isolates in [50], [52] . All other used data are the same as in figure 1 in the main text. (PDF) [file ppat.1004225.s013.pdf]

Acquired infections	estimate	p-value	lower 95% CI	upper 95% CI
Total incidence rate/ 1000 patient days	-4.70	0.037	-9.10	-0.29
Weighted incidence rate resistant infections / 1000 patient days	-5.14	0.095	-11.18	0.89
Deaths/1000 patient days	-1.68	0.045	-3.33	-0.04
