# Supplementary material for: Cycling Empirical Antibiotic Therapy in Hospitals: Meta-Analysis and Models
Source: PLoS Pathog. 2014 Jun 26;10(6):e1004225. doi: 10.1371/journal.ppat.1004225 (PMC4072793; doi:10.1371/journal.ppat.1004225)
Supplement: Table S5 — Results of meta-analyses under inclusion of the contemporary instead of the historic control arm in [45] . All other used data are the same as in figure 1 in the main text. (PDF) [file ppat.1004225.s014.pdf]

Contemporary control	estimate	p-value	lower 95% CI	upper 95% CI
Total incidence rate/ 1000 patient days	-4.54	0.055	-9.18	0.09
Weighted incidence rate resistant infections / 1000 patient days	-7.33	0.035	-14.14	-0.53
Deaths/1000 patient days	-1.43	0.078	-3.01	0.16
