# Supplementary material for: Cycling Empirical Antibiotic Therapy in Hospitals: Meta-Analysis and Models
Source: PLoS Pathog. 2014 Jun 26;10(6):e1004225. doi: 10.1371/journal.ppat.1004225 (PMC4072793; doi:10.1371/journal.ppat.1004225)
Supplement: Table S6 — Overview over parameters. A) Ward characteristics [73]. * The average length of stay is 8 days in Switzerland (http://www.obsandaten.ch/indikatoren/5_4_1/2005/d/541.pdf, data from 2005) and 5 days in the US (http://www.cdc.gov/mmwr/preview/mmwrhtml/mm5427a6.htm). ** Pennsylvania Health Care Cost Containment Council. Hospital-acquired Infections in Pennsylvania 2005, 2006 & 2007. Available: http://www.phc4.org/reports/hai/(2011). ***χ was chosen arbitrarily. However, βE is adjusted such that R0 remains constant for both direct and environmental transmission or any mixture of these two transmission modes. B) Composition of incoming patients [74]. C) Disease biology [1] [75]–[77] [78], [79] [80], [81]. * “Colonization pressure”, i.e. the frequency of both asymptomatic and symptomatic carriers in a hospital ward has been shown to be a major risk factor for the acquisition of a nosocomial pathogen [82]. It also has been shown for Clostridium difficile, that environmental contamination occurred for both symptomatic and asymptomatic infections [83]. Nevertheless, it is conceivable that e.g. in symptomatically infected patients with diarrhea infectivity is much higher than in asymptomatic patients. However, since the connection between carriage and infection is established and data on potential differences in infectivity between symptomatic and asymptomatic patients are scarce, we chose not to distinguish between these two classes. D) Treatment characteristics. (PDF) [file ppat.1004225.s015.pdf]

A

	Explanation	Screened range	Default
N	Number of beds	-	20
t_{LOS}	Length of stay for asymptomatic patients [days]	2, 5, 7, 10, 15, 20, 50	7 days*
r_I	Retention of infected patients (i.e. factor by which discharge of infected is more or less likely than for asymptomatic patients; low values indicate symptomatic infected are more likely to be removed, e.g. isolation; high values indicate symptomatic patients remain very likely in hospital)	0.5, 1, 3, 5, 10, 20, 50	3**
t_e	Half-life of pathogens in reservoir [days]	1, 2, 5, 10, 20, 30, 50	20 days [73]
χ	Rate of environmental contamination	-	0.1***

B

	Explanation	Screened range	Default
p_P	Proportion protecteds among incoming patients [%]	0, 15, 30, 45, 60, 75, 90	0%
p_S	Proportion patients that are not fully protected by microflora [%]	0, 20, 40, 60, 80, 95, 99	20%
f_i	Proportion of incoming carriers that are symptomatic [%]	0, 5, 20, 40, 60, 80, 100	5% (i.e. 0.25% of admitted patients symptomatic carriers)
f_{res}	Fraction of carriers (C +I) carrying strains resistant to either A, B or AB [%]	0, 10, 20, 40, 60, 80, 100	scenario i: 0%<br>scenario ii and iii: 10%<br>(i.e. 2% of admitted patients are carriers of a resistant strain)[74]
p_{asym}	Asymmetry of A- and B-resistance among incoming patients (low values indicate all B- or AB-resistant; high values indicate all A- or AB- resistant) [%]	0, 20, 40, 50, 60, 80, 100	50% (as many A- as B-resistant)
f_{double}	Fraction double-resistance of all resistant strains (low values indicate only single-resistances; high values indicate only double-resistance) [%]	0, 20, 40, 50, 60, 80, 100	scenario i and ii: 0%<br>scenario iii: 40%<br>(i.e. 0.8% of admitted patients are carriers of a double resistant strain)[74]

C

	Explanation	Screened range	Default
c_a	cost of resistance A [%]	0, 10, 20, 30, 40, 50, 60	10% [75-77]
c_b	cost of resistance B [%]	0, 10, 20, 30, 40, 50, 60	10% [75-77]
c_{ab}	cost of resistance AB [%]	0, 10, 20, 30, 40, 50, 60	20% [75-77]
$t_{cl,P}$	Time until protection by microflora is gone when treated with broadspectrum	-	-
t_{cl}	Time until carrier is not infectious when appropriately treated [days]	1, 2, 5, 7, 10, 15, 20	5 days [78, 79]
μ_a	Frequency of new A-resistance mutation per patient day	10^{-1}, 10^{-2}, 10^{-3}, 10^{-4}, 10^{-5}, 10^{-6}, 10^{-7}	10^{-4} (both A and B equally likely)
μ_a/μ_b	Relative frequency of new B-resistance mutations per day	10^{-2}, 10^{-1}, 1, 10, 10^{2}, 10^{4}, 10^{6}	1 (both A and B equally likely)
$\mu_{ab}/(\mu_a*\mu_b)$	Relative frequency of new AB-resistance mutations per day	10^{-3}, 10^{-2}, 10^{-1}, 1, 10, 10^{2}, 10^{3}	1 (both A and B mutations independent)
d	Time to death if untreated [days]	2, 5, 10, 20, 30, 50, 100	7 days
t_P	Time to progression from colonized to infected [days]	1, 5, 7, 10, 20, 50, never	7 days (this corresponds to ~8% of patients acquiring an infection in the hospital, which is global average) [1]
β_D	Infectious encounters between patients resulting in colonization per patient day (calculated for 20 beds)	0.005, 0.01, 0.015, 0.02, 0.025, 0.03, 0.035	0.02 [22, 23] (R_0 of wild-type ≈ 1.5, R_A, the number of secondary infections in a hospital is ~0.4, because we assume that there is no superinfection and hence only a fraction of the patients is susceptible)*
β_E/β_D	Infectious encounters with environment resulting in coloni-zation per patient day (calculated for 20 beds) relative to β_D	0, 0.17, 0.33, 0.50, 0.67, 0.83, 1	Equivalent to $\beta_D/t_e\chi$ (adjusted such that for any given environmental decay rate t_e, R_0 of environmental transmission is equal to the R_0 for direct transmission in the same scenario)

D

	Explanation	Screened range	Default
t_s	Rate with which inappropriate treatment of infected patients is switched from A to B or B to A. This is not the same as colonized patients progressing to symptomatic disease, in which case we always assume that treatment is switched within a day	never, 0.17, 0.33, 0.50, 0.67, 0.83, 1	never
f_p	fraction of asymptomatic patients receiving scheduled treatment for prophylaxis or treatment of other disease [%]	0, 5, 10, 15, 20, 30, 40	20%
$f_{p,AB}$	fraction of asymptomatic patients additionally receiving both drug A and B for prophylaxis or treatment of other disease [%]	0, 1, 5, 10, 20, 50, 100	1%
