# Supplementary material for: Cycling Empirical Antibiotic Therapy in Hospitals: Meta-Analysis and Models
Source: PLoS Pathog. 2014 Jun 26;10(6):e1004225. doi: 10.1371/journal.ppat.1004225 (PMC4072793; doi:10.1371/journal.ppat.1004225)
Supplement: Text S1 — Detailed description of meta-analysis (method, extracted data and sensitivity analysis) and sensitivity analysis of theoretical model. (DOCX) [file ppat.1004225.s016.docx]

**Supplemental Text S1**

**Cycling empirical antibiotic therapy in hospitals: meta-analysis and models**

Pia Abel zur Wiesch*^,#,1,2^, Roger Kouyos^#,1,3^, Sören Abel^4^, Wolfgang Viechtbauer^5^ and Sebastian Bonhoeffer^1^

^1^ Institute of Integrative Biology, ETH Zurich, Zurich, Switzerland

^2^ Division of Global Health Equity, Brigham and Women`s Hospital/Harvard Medical School, Boston, MA, USA

^3^ Division of Infectious Diseases and Hospital Epidemiology, University Hospital Zurich, Zurich, Switzerland

^4^ Division of Infectious Diseases, Brigham and Women`s Hospital/Harvard Medical School, Boston, MA, USA

^5^ Department of Psychiatry and Psychology, School for Mental Health and Neuroscience, Faculty of Health, Medicine, and Life Sciences, Maastricht University, Maastricht, The Netherlands

^#^ Authors contributed equally to the manuscript

*** Corresponding author:**Pia Abel zur Wiesch
Division of Global Health Equity,
Brigham and Women`s Hospital/Harvard Medical School
641 Huntington Avenue
MA-02115 Boston, USA
pzw@daad-alumni.de

**Running title:** Cycling empirical antibiotic therapy

**Table of contents**

**Meta-Analysis p. 3**

*Random-Effects Multivariate Meta-Analysis Model p. 3*

*Comparison to univariate meta-analysis p. 4*

*Results of meta-regression p. 4*

*Sensititivity-analysis meta-analysis p. 4*

**Sensitivity Analysis Theoretical Model p. 5**

*Differences between stochastic and deterministic model and*

*environmental vs. direct transmission p. 5*

*Factors influencing optimal period length p. 5*

**References p. 8**

*Random-Effects Multivariate Meta-Analysis Model*

The three endpoints (i.e., the incidence rate difference for the number of total isolates, for the number of resistant isolates, and for mortality) were analyzed simultaneously using a random-effects multivariate meta-analysis model along the lines of multivariate meta-analytic models described previously in the literature [[1-3](#_ENREF_1)]. The basic model is given by $y_{ij}=\mu_{j}+u_{ij}+e_{ij}$, where $y_{ij}$ is the observed incidence rate difference in study $i$ for endpoint $j$ ($i=1,\ldots,12$ and $j=1,2,3$), $\mu_{j}$ is the true average value of the incidence rate difference for endpoint $j$, $u_{ij}\sim MVN(0,G)$ are multivariate normally distributed random effects to model heterogeneity in the true values for the three endpoints and to account for their non-independence, and $e_{ij}\sim MVN(0,S_{i})$ are multivariate normally distributed sampling errors. In matrix notation, the model can be written as:

$\left[ \begin{matrix} y_{11} \\ y_{12} \\ y_{13} \\ y_{21} \\ y_{22} \\ y_{23} \\ \vdots\end{matrix} \right]=\left[ \begin{matrix} 1 & 0 & 0 \\ 0 & 1 & 0 \\ 0 & 0 & 1 \\ 1 & 0 & 0 \\ 0 & 1 & 0 \\ 0 & 0 & 1 \\ \vdots& \vdots& \vdots\end{matrix} \right]\left[ \begin{matrix} \mu_{1} \\ \mu_{2} \\ \mu_{3} \end{matrix} \right]$+$\left[ \begin{matrix} u_{11} \\ u_{12} \\ u_{13} \\ u_{21} \\ u_{22} \\ u_{23} \\ \vdots\end{matrix} \right]+\left[ \begin{matrix} e_{11} \\ e_{12} \\ e_{13} \\ e_{21} \\ e_{22} \\ e_{23} \\ \vdots\end{matrix} \right]$

where

$$Var\left[ \begin{matrix} u_{i1} \\ u_{i2} \\ u_{i3} \end{matrix} \right]=G=\left[ \begin{matrix} \tau_{1}^{2} & \rho_{12}\tau_{1}\tau_{2} & \rho_{13}\tau_{1}\tau_{3} \\ & \tau_{2}^{2} & \rho_{23}\tau_{2}\tau_{3} \\ & & \tau_{3}^{2} \end{matrix} \right]$$

and

$$Var\left[ \begin{matrix} e_{i1} \\ e_{i2} \\ e_{i3} \end{matrix} \right]=S_{i}=\left[ \begin{matrix} \sigma_{i1}^{2} & \gamma_{i12}\sigma_{i1}\sigma_{i2} & \gamma_{i13}\sigma_{i1}\sigma_{i3} \\ & \sigma_{i2}^{2} & \gamma_{i23}\sigma_{i2}\sigma_{i3} \\ & & \sigma_{i3}^{2} \end{matrix} \right]$$

and independence is assumed between studies (only the upper triangular part of $G$ and $S_{i}$ are shown). For studies not providing data for all three endpoints, the rows/columns corresponding to the non-reported endpoint(s) are deleted.

The sampling variances (i.e., the diagonal elements in $S_{i}$) can be computed for each study using known properties of Poisson distributed random variables [[4](#_ENREF_4)] but none of the studies provided sufficient information to compute the correlation (and hence, the covariance) among the sampling errors (i.e., $\gamma_{i12}$, $\gamma_{i13}$, $\gamma_{i23}$), a common issue in multivariate meta-analyses. We therefore substituted a single correlation parameter for the sampling errors across all studies, so that

$$Var\left[ \begin{matrix} e_{i1} \\ e_{i2} \\ e_{i3} \end{matrix} \right]=S_{i}=\left[ \begin{matrix} \sigma_{i1}^{2} & \gamma\sigma_{i1}\sigma_{i2} & \gamma\sigma_{i1}\sigma_{i3} \\ & \sigma_{i2}^{2} & \gamma\sigma_{i2}\sigma_{i3} \\ & & \sigma_{i3}^{2} \end{matrix} \right]$$

and then simultaneously estimated $\gamma$ along with the other model parameters using restricted maximum likelihood (REML) estimation (an approach similar to the Riley model [[5](#_ENREF_5),[6](#_ENREF_6)], but without assuming that the correlation among the sampling errors and the true effects is identical). We checked for model identifiability by profiling each parameter (i.e., in turn, we fixed each parameter within a range of values around its estimate, then maximized the restricted log likelihood over the remaining parameters, and then ascertained that each profile likelihood function is concave, smooth, and shows a peak around its estimate).

*Comparison to univariate meta-analysis*

In univariate analyses, cycling was beneficial in reducing all endpoints (Table S3).

*Results of meta-regression*

We found a total of 3 significant associations of study characteristics with outcome: i) as discussed in the main text, the reduction in the incidence rate of total infections is correlated with baseline resistance levels

ii) The reduction in mortality was correlated with the ratio of antibiotic consumption between the cycling and mixing arm. The lower the antibiotic consumption in cycling relative to baseline, the more pronounced the reduction in mortality. However, it is difficult to establish causality here, because such a finding could also be due to fewer critically ill patients in the cycling arm, who then would require fewer antibiotics.

iii) The benefit of clinical cycling in reducing resistance was correlated with the ratio of antibiotic heterogeneity indices (AHIs [[7](#_ENREF_7)]) during a single cycling period and during baseline. For example, if adherence was 100% and only one drug is employed during each cycle, the AHI would be 0, while the AHI of 50/50 mixing of two drugs would be 1. While adherence will never reach 100%, we would still expect heterogeneity to be higher during the baseline than in each cycling period- this is not always true in the studies collected here. Only three studies gave sufficient data to assess this criterion, additionally, there was a large variance in how comprehensively antibiotic usage was reported. Although we would expect that “adjustable cycling” is beneficial if the ratio of heterogeneities is low, both the variability in reported data and the low number of studies make conclusions difficult.

*Sensititivity-analysis meta-analysis*

One study [[8](#_ENREF_8)] only reports infections with strains that patients were neither colonized nor infected with when admitted (i.e., strains were acquired in hospital). Two other studies [[9](#_ENREF_9),[10](#_ENREF_10)] report both total infections and acquired infections. For the sake of consistency with the other studies, we use here only total infections when reported. We also performed the same meta-analysis for the total incidence rate, resistant incidence rate and mortality with acquired infections in [[9](#_ENREF_9),[10](#_ENREF_10)]. The reduction in total infections remained significant and the reduction in mortality became significant (Table S4).

One study [[11](#_ENREF_11)] provides data on both a historic baseline in the same ward and a contemporary control arm in a comparable ward in the same hospital. For the sake of consistency with all but one of the other studies, we use here the historic baseline as control. We also performed the same meta-analysis for the total incidence rate, resistant incidence rate and mortality with the contemporary control arm in [[11](#_ENREF_11)]. The reduction in resistant infections remained significant (Table S5).

Since not all sensitivity analyses are available yet for multi-variate models, we chose the univariate models to test for outliers by leave-one-out and influence analyses. We performed three meta-analyses (total incidence rate, weighted incidence rate of resistant infections and death rate) and show one meta-regression (total incidence rate vs. baseline resistance levels). To address the influence of single studies on these results, we used the function influence() in the package metafor of the statistical package R [[12](#_ENREF_12)]. This function returns leave-one-out diagnostics. According to these criteria, none of the studies in our analysis for total incidence rate or incidence rate of resistant infections was classified as an outlier. We also performed a “leave-one-out” analysis of the meta-analysis results for the total incidence rate, resistant incidence rate (data not shown). In none of these, the sign of the prediction changed, i.e. cycling remained beneficial. However, the confidence intervals widened.

**Sensitivity Analysis Theoretical Model**

*Differences between stochastic and deterministic model and environmental vs. direct transmission*

As mentioned in the main part, stochastic models generally perform somewhat worse because emergence of resistance is less likely due to extinction events (compare Figure 3 to Figure S2 and S3). Compared to direct transmission, environmental transmission leads to fewer extinction events in the stochastic model. This allows for more emergence of double resistance, leading to a better performance of cycling.

*Factors influencing optimal period length*

As discussed in the main text, we screened a very large parameter space. For each unique parameter set, we give the optimal period length and its effect for all standard scenarios (Figure S4). We analyzed how each factor individually changed the relationship of total infections, inappropriately treated patients, and genotype composition with the period length (data not shown). Our parameter screen revealed that three main factors influence the optimal period length as well as the success when employing this period: i) the prevalence of the different genotypes among incoming patients, ii) the generation time (i.e. the time between the colonization of a patient and the transmission of the pathogen) as well as iii) the competitive fitness of the different genotypes. These three factors are discussed in detail below.

In most scenarios, our model assumes a constant influx of resistant strains into the ward. Thus, the frequency of the strains is determined by both the influx of this strain and its ability to transmit. The essential difference between cycling and mixing is that the prevalence of each single-resistant strain fluctuates strongly, depending on which drug is currently used (Figure 2). With a constant and high supply of singly resistant strains these fluctuations are dampened, because transmission within the ward becomes unimportant compared to the influx from outside. In this case, the performance of “adjustable cycling” and “adjustable mixing” becomes more similar. This is both true for short cycling periods, where “adjustable cycling” can be beneficial, as well as for long periods, where “adjustable cycling” is strongly detrimental (Figure S5A).

When more infected or colonized patients are admitted to the hospital, also more patients with resistant infections come into the hospital. With a constant and high supply of singly resistant strains, there are little differences between “adjustable cycling” and “adjustable mixing”. The same is true when only few susceptible patients are admitted, since the currently favored strains cannot be transmitted.

Another important factor is the generation time, i.e. the time between the infection of a patient and the transmission of the pathogen to the next patient. With environmental transmission, the generation time is determined by the environmental decay rate and not by patient turnover. As mentioned above, cycling periods below the generation time essentially result in mixing. Thus, the exact period in which “adjustable cycling” is most beneficial increases with the generation, i.e. length of stay or the duration of pathogen persistence in the environment (Figure S5B).

Our model predicts that “adjustable cycling” develops its full benefits in large populations where the emergence of multiple resistance is unavoidable or where occasionally imported strains do not immediately go extinct. This is true for most cases unless the mutation rate for double resistance becomes so high (extremes of the screened range) that even cycling cannot suppress multiple resistance. We see comparable effects in the stochastic version that describes small wards (Figure S4): A high probability of acquiring multiple resistance makes “adjustable cycling” especially successful. This is true when either the rate of double-resistance acquisition is high or the stepwise acquisition of double resistance is fast (i.e. both mutation rates are equal, such that their product is maximal). At the same time, low mutation rates for single resistance have the same effect. This is because at any single time-point during cycling, the double-resistant strain has no immediate advantage over the currently untreated single-resistant strain. If double-resistance is more costly than single-resistance, the double-resistant strain is then at a disadvantage. Clearly, this only makes “adjustable cycling” more beneficial if “adjustable mixing” fails to suppress the rise of double-resistance, i.e. the double-resistant strain is as least as fit as the single resistant strain during mixing (see Figure S6). In other words, cycling can only be beneficial at intermediate fitness costs of double resistance relative to the single resistant strain, that is if the double-resistant strain is less fit than the single resistant during a single cycling period, but also fit enough to be competitive during mixing.

The suppression of multiple resistance becomes more and more pronounced with increasing periods. Thus, a higher probability of emergence of double resistance does not only increase the success of “adjustable cycling”, it also shifts the optimal period towards higher values. When double-resistance is unlikely to emerge, such as in the standard setting of our stochastic model, “adjustable cycling” only confers the advantage of lowering the level of single resistance for periods slightly longer than the turnover rate. If avoiding inappropriate empirical therapy is the main concern, it might therefore pay off to outcompete double-resistant strains even at the cost of increased levels of single-resistance.

Figure S7 gives an overview of the three factors influencing whether and at which period length “adjustable cycling” outperforms mixing. While the protection by a microflora plays an important role in the general dynamics of resistance spread [[13](#_ENREF_13)], this factor (as modeled here by the patient compartment “P”) has no influence on the relative success of “adjustable cycling” in our model.

**References**

1. Kalaian HA, Raudenbush SW (1996) A multivariate mixed linear model for meta-analysis. Psychological Methods 1: 227-235.

2. Berkey CS, Hoaglin DC, Antczak-Bouckoms A, Mosteller F, Colditz GA (1998) Meta-analysis of multiple outcomes by regression with random effects. Stat Med 17: 2537-2550.

3. Riley RD, Abrams KR, Lambert PC, Sutton AJ, Thompson JR (2007) An evaluation of bivariate random-effects meta-analysis for the joint synthesis of two correlated outcomes. Stat Med 26: 78-97.

4. Bagos PG, Nikopoulos GK (2009) Mixed-effects Poisson regression models for meta-analysis of follow-up studies with constant or varying durations. The International Journal of Biostatistics 5.

5. Riley RD, Thompson JR (2008) An alternative model for bivariate random-effects meta-analysis when the within-study correlations are unknown. Biostatistics 9: 172-186.

6. Riley RD (2009) Multivariate meta-analysis: The effect of ignoring within-study correlation. Journal of the Royal Statistical Society, Series A 178: 789-811.

7. Sandiumenge A, E. Diaz, A. Rodriguez, et al. (2006) Impact of diversity of antibiotic use on development of antimicrobial resistance. Journal of Antimicrobial Chemotherapy 57: 1197-1204.

8. Smith RL, H.L. Evans, T.W. Chong, et al. (2008) Reduction in rates of methicillin-resistant Staphylococcus aureus infection after introduction of quarterly linezolidvancomycin cycling in a surgical intensive care unit. Surgical Infections 9: 423-431.

9. Nijssen S, A. Fluit, D. van de Vijver, et al. (2010) Effects of reducing beta-lactam antibiotic pressure on intestinal colonization of antibiotic-resistant gram-negative bacteria. Intensive Care Medicine 36: 512-519.

10. Hedrick TL, A.S. Schulman, S.T. McElearney, et al. (2008) Outbreak of resistant Pseudomonas aeruginosa infections during a quarterly cycling antibiotic regimen. Surgical Infections 9: 139-152.

11. Bennett KM, J.E. Scarborough,M. Sharpe, et al. (2007) Implementation of antibiotic rotation protocol improves antibiotic susceptibility profile in a surgical intensive care unit. Journal of Trauma 63: 307-311.

12. Viechtbauer W (2010) Conducting meta-analyses in R with the metafor package. Journal of Statistical Software 36: 1–48.

13. Lipsitch M, Bergstrom CT, Levin BR (2000) The epidemiology of antibiotic resistance in hospitals: paradoxes and prescriptions. Proc Natl Acad Sci U S A 97: 1938-1943.
